# FOXO1 is a master regulator of CAR T memory programming

**DOI:** 10.21203/rs.3.rs-2802998/v1

**Published:** 2023-11-07

**Authors:** Alexander Doan, Katherine P. Mueller, Andy Chen, Geoffrey T. Rouin, Bence Daniel, John Lattin, Yingshi Chen, Brett Mozarsky, Martina Markovska, Jose Arias-Umana, Robert Hapke, Inyoung Jung, Peng Xu, Dorota Klysz, Malek Bashti, Patrick J Quinn, Katalin Sandor, Wenxi Zhang, Junior Hall, Caleb Lareau, Stephan A. Grupp, Joseph A. Fraietta, Elena Sotillo, Ansuman T. Satpathy, Crystal L. Mackall, Evan W. Weber

**Affiliations:** 1. Center for Cancer Cell Therapy, Stanford Cancer Institute, Stanford University School of Medicine, Stanford, CA 94305, USA; 2. Department of Pediatrics, Division of Oncology, Perelman School of Medicine, University of Pennsylvania, Philadelphia, PA 19104 USA; 3. Center for Childhood Cancer Research, The Children’s Hospital of Philadelphia, Philadelphia, PA 19104, USA; 4. Raymond G. Perelman Center for Cellular and Molecular Therapeutics, The Children’s Hospital of Philadelphia, Philadelphia, PA, 19104, USA; 5. Abramson Cancer Center, Perelman School of Medicine, University of Pennsylvania, Philadelphia, PA 19104, USA.; 6. Center for Cellular Immunotherapies, Perelman School of Medicine, University of Pennsylvania, Philadelphia, PA 19104, USA.; 7. Department of Pathology, Stanford University, Stanford, CA 94305, USA; 8. Department of Bioengineering, Stanford University, Stanford, CA 94305, USA; 9. Gladstone-UCSF Institute of Genomic Immunology, San Francisco, CA 94158, USA; 10. Cell and Molecular Biology Graduate Program, Perelman School of Medicine, University of Pennsylvania, Philadelphia, PA, USA.; 11. Center for Personal Dynamic Regulomes, Stanford University, Stanford, CA 94305, USA; 12. Department of Microbiology, Perelman School of Medicine, University of Pennsylvania, Philadelphia, PA 19104, USA; 13. Parker Institute for Cancer Immunotherapy, San Francisco, CA 94129 USA; 14. Department of Microbiology, Perelman School of Medicine, University of Pennsylvania, Philadelphia, PA 19104, USA; 15. Department of Pathology and Laboratory Medicine, Perelman School of Medicine, University of Pennsylvania, Philadelphia, PA 19104, USA; 16. Division of Hematology, Oncology, Stem Cell Transplantation and Regenerative Medicine, Department of Pediatrics, Stanford University, Stanford, CA 94305, USA; 17. Division of Blood and Marrow Transplantation and Cell Therapy, Department of Medicine, Stanford University, Stanford, CA 94305, USA

## Abstract

Poor CAR T persistence limits CAR T cell therapies for B cell malignancies and solid tumors^1,2^. The expression of memory-associated genes such as *TCF7* (protein name TCF1) is linked to response and long-term persistence in patients^3–7^, thereby implicating memory programs in therapeutic efficacy. Here, we demonstrate that the pioneer transcription factor, FOXO1, is responsible for promoting memory programs and restraining exhaustion in human CAR T cells. Pharmacologic inhibition or gene editing of endogenous *FOXO1* in human CAR T cells diminished the expression of memory-associated genes, promoted an exhaustion-like phenotype, and impaired antitumor activity *in vitro* and *in vivo*. FOXO1 overexpression induced a gene expression program consistent with T cell memory and increased chromatin accessibility at FOXO1 binding motifs. FOXO1-overexpressing cells retained function, memory potential, and metabolic fitness during settings of chronic stimulation and exhibited enhanced persistence and antitumor activity *in vivo*. In contrast, TCF1 overexpression failed to enforce canonical memory programs or enhance CAR T cell potency. Importantly, endogenous FOXO1 activity correlated with CAR T and TIL responses in patients, underscoring its clinical relevance in cancer immunotherapy. Our results demonstrate that memory reprogramming through FOXO1 can enhance the persistence and potency of human CAR T cells and highlights the utility of pioneer factors, which bind condensed chromatin and induce local epigenetic remodeling, for optimizing therapeutic T cell states.

Chimeric antigen receptor T cell (CAR T) therapies have exhibited remarkable response rates in patients with B cell malignancies who are refractory to all other forms of therapy. However, over 50% of responding patients eventually relapse, and CAR T cells targeting solid tumors have been largely ineffective. A major factor limiting CAR T therapy is poor CAR T cell persistence, which can lead to less durable antitumor activity in patients, incomplete tumor regression, or relapsed disease. Recent work interrogating the transcriptome of pre-manufacturing, pre-infusion or post-infusion CAR T cells demonstrated that genes associated with T cell memory correlate with complete response and long-term CAR T persistence (≥ 6 months of B cell aplasia), whereas genes associated with T cell exhaustion correlated with partial or non-response and short-term persistence^3–7^. In particular, the memory-associated transcription factor *TCF7* (protein name TCF1) broadly correlates with response to CAR T^3,6^, Tumor-infiltrating lymphocyte (TIL)^8^, and checkpoint blockade therapy responses in patients^9,10^. Despite evidence that indirect induction of *TCF7* expression can endow T cells with stem-like properties that promote antitumor activity^11–14^, the direct therapeutic relevance of *TCF7* in CAR T cells is not well-established.

We previously demonstrated that providing a period of rest to exhausted CAR T cells through transient inhibition of CAR signaling restored antitumor functionality, promoted a memory-like phenotype, and led to increased chromatin accessibility at motifs bound by the memory TFs *TCF7* and *FOXO1*^15^. FOXO1 directly regulates the expression of *TCF7* and other canonical memory genes, including *SELL*^16^ and *IL7R*^17^, and promotes formation of central memory or *TCF7*-expressing progenitor exhausted T cells (T_PEX_) during acute and chronic infection, respectively^17–20^, raising the prospect that FOXO1 and/or TCF1 are primary drivers of memory T cell programming. Consistent with this notion, FOXO1-associated gene signatures (ex. STAT3 and IL-6) correlate with CAR T responses in patients^3^, and indirect pharmacologic activation of FOXO1 *in vitro* can improve human CAR T cell function^21^. However, the clinical relevance of endogenous *FOXO1* in human T cells and the extent to which TCF1 and/or FOXO1 induce memory programming and function in CAR T cells remains unclear.

Here, we demonstrate that FOXO1 is required for memory programming, and its overexpression enhances the persistence and potency of human CAR T cells. In contrast, TCF1 overexpression induces a T_PEX_-like transcriptional program and fails to enhance antitumor activity, challenging the notion that TCF1 is therapeutically relevant in CAR T cell therapy. Collectively, these studies implicate T cell memory gene expression programs as a core determinant of CAR T function in preclinical models and patients, suggesting that memory reprogramming via transcription factor engineering may represent a universal strategy to enhance CAR T cell efficacy.

## Endogenous FOXO1 promotes memory and restrains exhaustion in human CAR T cells

**Extended Data Fig 1:**
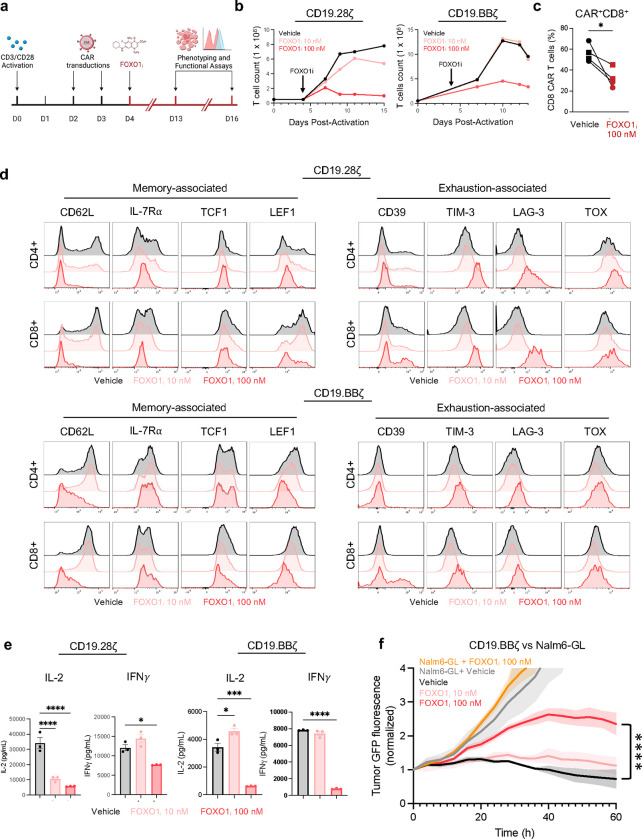
Pharmacologic inhibition of FOXO1 impairs expansion, formation of a memory phenotype, and function in CD19.28ζss and CD19.BBζ CAR T cells. CAR T cells were treated with DMOS (vehicle, black) or 10nM (pink) or 100nM (red) of the small molecule inhibitor AS1842856 (FOXO1_i_) starting on day 4 post-activation and treated every 2–3 days thereafter. Phenotypic and functional assays were performed between day 13 and day 16. **a,** Schematic of FOXO1_i_ experimental model. **b,** T cell expansion kinetics of CD19.28ζ (right) or CD19.BBζ (right) CAR T cells. **c,** Percent CD8+ cells in CD19.28ζ and CD19.BBζ CAR T cells (*n* = 2 donors for CD19.28ζ, circles, and CD19.BBζ, squares). **d,** Expression of memory- (left) and exhaustion-associated markers (right) on CD19.28ζ (top) and CD19.BBζ (bottom) CAR T cells. Histograms from a representative donor are shown. **e,** IL-2 and IFNγ secretion from CD19.28ζ (left) and CD19.BBζ (right) in response to Nalm6 leukemia cells. Graphs show mean ± s.d. of triplicate wells from a representative donor (*n* = 2 donors). **f,** Cytotoxicity of CD19.BBζ CAR T cells (1:1 E:T, normalized to *t* = 0). Graph shows mean ± s.d. of triplicate wells from a representative donor (*n* = 2 donors). **c**, Paired two-sided student’s t-test; **e**, 1-way ANOVA with Dunnett’s multiple comparisons test; **f,** 2-way ANOVA with Dunnett’s multiple comparisons at *t* = 60 hours. *, *P* < 0.05; ***, *P* < 0.001; ****, *P* < 0.0001.

Because FOXO1 target gene expression and associated pathways correlate with clinical CAR T cell responses^3,6^, we sought to determine whether FOXO1 is required for memory programming and antitumor function in human CAR T cells. We cultured CD19.28ζ or CD19.BBζ CAR T cells in the presence of a selective FOXO1 small molecule inhibitor^22^ (FOXO1_i_) for approximately 10 days and subsequently performed phenotypic and functional experiments on day 15 (Extended Data Fig. 1a). FOXO1_i_ dose-dependently reduced CAR T expansion, CD8^+^ frequency, and expression of memory-associated markers (CD62L, IL-7Rα, TCF1, and LEF1), and concomitantly upregulated markers that demarcate short-lived or exhausted T cells (CD39, TIM-3, LAG-3, TOX) (Extended Data Fig. 1b–d).

**Extended Data Fig 2:**
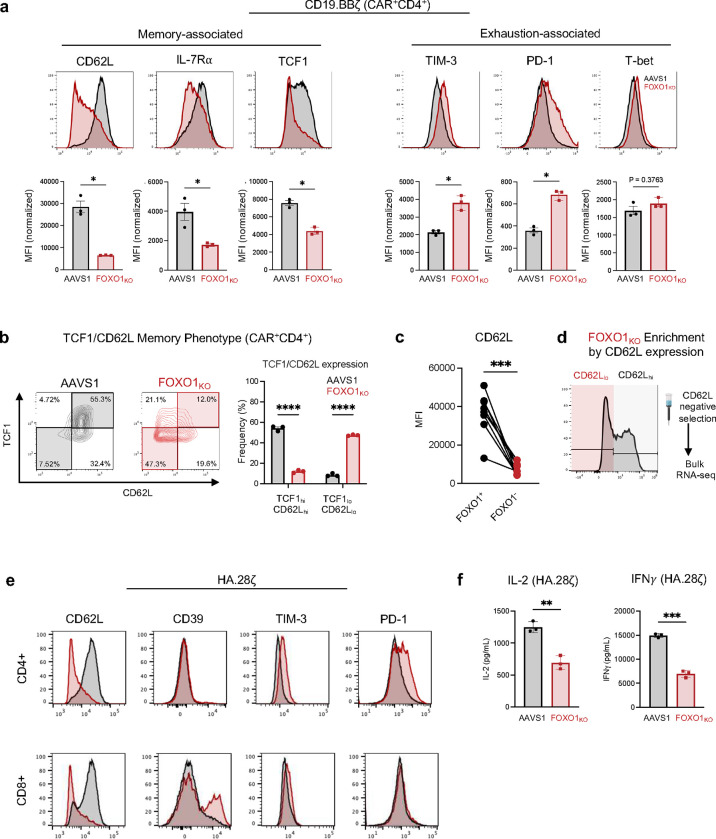
CRISPR knockout of FOXO1 promotes an exhausted phenotype in CD4+ CD19.BBζ and in HA.28ζ CAR T cells. **a,** Expression of memory- (left) and exhaustion-associated markers (right) on CD4^+^ CD19.BBζ CAR T cells with AAVS1 gene-editing (black) or FOXO1_KO_ (red). Histograms show a representative donor and bar graphs show mean ± s.e.m. of 3 donors (CD8+ cells appear in Fig. 1). **b,** TCF1 and CD62L expression in CD4+ CD19.BBζ CAR T cells. Contour plots show a representative donor and bar graphs show mean ± s.e.m. of 3 donors (CD8^+^ cells appear in Fig. 1). **c,** Mean fluorescence intensity (MFI) of CD62L in FOXO1+ and FOXO1− gated subpopulations of CD19.BBζ CAR T cells at Day 21. **d,** Schematic showing CD62L_lo_ / FOXO1_KO_ cell negative selection strategy for RNA-sequencing experiments (Fig. 1f,g). **e,** Expression of memory- and exhaustion-associated markers on day 15 HA.28ζ CAR T cells **f,** IL-2 (left) and IFNγ (right) secretion from HA.28ζ CAR T cells in response to Nalm6 leukemia. Graphs show one representative donor (*n* = 2 donors). **a,c,** Paired two-sided student’s t-test; **b,** 2-way ANOVA with Bonferroni’s multiple comparisons test; **f,** Welch’s T-test. *, *P* < 0.05; **, *P* < 0.01; ***, *P* < 0.001; ****, *P* < 0.0001.

**Figure 1:**
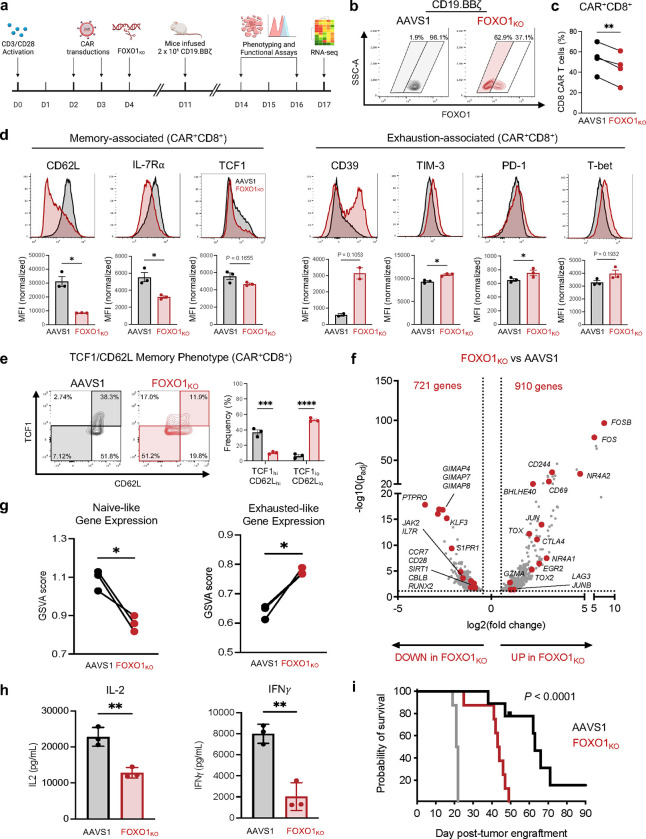
CRISPR knockout of human *FOXO1* impairs memory formation and antitumor function in CAR T cells. **a**, Schematic depicting generation of *FOXO1* knockout (FOXO1_KO_) CD19.BBζ CAR T cells and downstream assays. **b,** Gating strategy for flow cytometric analysis of *AAVS1*-edited controls (AAVS1) and FOXO1_KO_ cells. All samples were gated on live CAR^+^ cells. AAVS1 cells (left) were analyzed regardless of FOXO1 expression level (gray shading) and FOXO1_KO_ cells (right) were gated on the FOXO1 negative subpopulation (red shading). **c,** Percent CD8^+^ AAVS1 and FOXO1_KO_ CD19.BBζ CAR-T cells at day 16 post-activation (*n* = 4 independent donors). **d,** Flow cytometric analysis of memory- (left) and exhaustion-associated markers (right) on CD8^+^ CD19.BBζ CAR T cells edited for AAVS1 (black) FOXO1 (red) and gated as in (**b**). Histograms show a representative donor; bar graphs depict mean ± s.e.m. of 3 independent donors. **e,** Contour plots showing memory marker expression and frequency in CD8^+^ CD19.BBζ CAR T cells edited for AAVS1 (black) or FOXO1 (red) and gated as in (**b**). Representative contour plots from one donor show TCF1_hi_CD62L_hi_ (memory) and TCF1_lo_CD62L_lo_ (effector) subpopulations; bar graphs show mean ± s.e.m. for 3 independent donors. **f,** Volcano plot of differentially expressed genes (DEGs) in CD62L_lo_ FOXO1_KO_ versus AAVS1 CD19.BBζ CAR T cells from 3 independent donors (adjusted *P* < 0.05 with log2(fold change) > 0.5). Genes of interest are highlighted in red. **g,** Gene set variation analysis (GSVA) using naive and exhausted T cell gene signatures from Andreatta et al^66^. **h,** Day 14 IL-2 and IFNγ secretion in response to Nalm6 leukemia. Error bars represent mean ± s.d. of triplicate wells from one representative donor (*n* = 4 independent donors). **i,** Kaplan-Meier curves depicting survival of mice challenged with Nalm6 and treated with mock (grey), AAVS1 (black), or FOXO_KO_ (red) CD19.BBζ CAR T cells. Two independent donors were tested with 3–5 mice per group (*n* = 8 mock or FOXO1_KO_ mice, *n* = 9 AAVS1 mice). **c,d,g,** Paired two-sided student’s t-test. **e,** Two-way ANOVA with Bonferroni’s multiple comparisons test. **h,** Unpaired two-sided Welch’s t-test. **i,** Log-rank Mantel-Cox test. *, *P* < 0.05; **, *P* < 0.01; ***, *P* < 0.001; ****, *P* < 0.0001.

We corroborated these data by performing CRISPR/Cas9 gene-editing to knock out *FOXO1* expression (FOXO1_KO_) (Fig. 1a,b). FOXO1_KO_ CAR T cells exhibited a similar reduction in CD8^+^ frequency, diminished memory-associated markers and increased exhaustion-associated markers compared to AAVS1 controls (Fig. 1c–e and Extended Data Fig. 2a,b). Since FOXO1_KO_ cells uniformly exhibited low CD62L surface expression, we used CD62L as a surrogate marker for *FOXO1* editing and applied magnetic bead negative selection to enrich for CD62L_lo_/FOXO1_KO_ cells before performing bulk RNA-sequencing (Extended Data Fig. 2c,d). FOXO1_KO_ cells upregulated activation- and exhaustion-associated genes (*FOS*, *JUN*, *NR4A1/2*, *TOX*, *CD69*), downregulated memory and FOXO1 target genes (*IL7R, CCR7, KLF3)* (Fig. 1f), and exhibited less naive- and more exhausted-like gene expression signatures (Fig. 1g), consistent with a model wherein *FOXO1* restrains exhaustion and/or terminal differentiation in human T cells, similar to reports in mice^19,20,23^. FOXO1_i_ and FOXO1_KO_ cells also displayed attenuated killing and/or cytokine secretion after tumor challenge (Fig 1h, Extended Data fig. 1e,f). We corroborated these results using an *in vitro* CAR T exhaustion model (HA.28ζ CAR), whereby antigen-independent tonic CAR signaling induces features of exhaustion within approximately 1 week^15,24^. *FOXO1* knockout in HA.28ζ CAR T cells accelerated acquisition of the exhausted phenotype and loss of function (Extended Data Fig. 2e,f). We next modeled chronic antigen stimulation *in vivo* by infusing a sub-therapeutic dose of CD19.BBζ CAR T cells into NOD/SCID/IL2Rg^−/−^ (NSG) mice bearing high burden Nalm6 leukemia^15,25^. Consistent with *in vitro* data, loss of *FOXO1* significantly reduced CAR T tumor control and survival (Fig 1i). Together, these observations demonstrate that in human T cells, endogenous *FOXO1* promotes a memory phenotype, restrains exhaustion, and is required for optimal CAR T cell antitumor function.

## FOXO1 overexpression preserves a memory phenotype and enhances antitumor function and metabolic fitness during chronic antigen stimulation

**Extended Data Fig 3:**
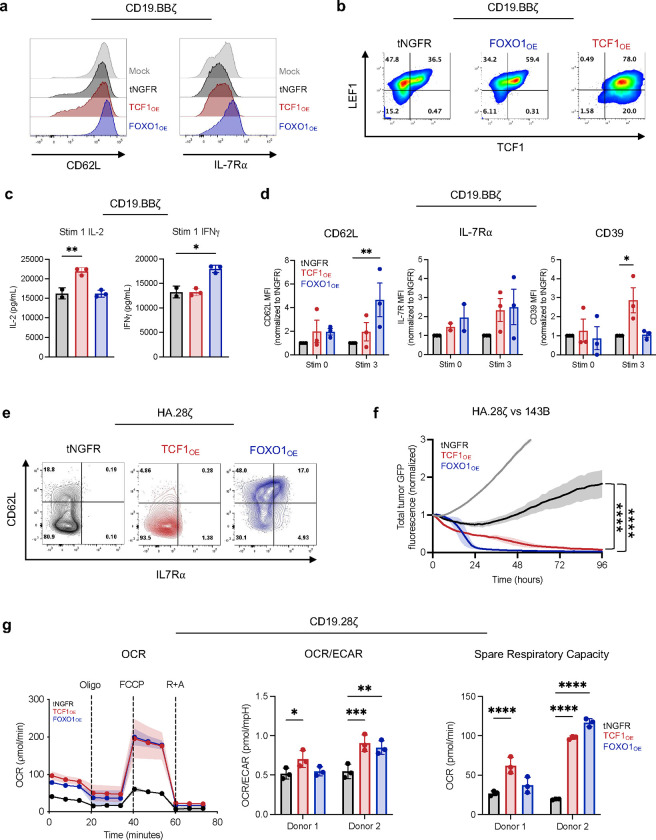
FOXO1 overexpression promotes a memory phenotype and mitigates exhaustion in CAR T cells. **a,** CD62L and IL-7Rɑ expression in CD19.BBζ CAR T cells from one representative donor (*n* = 4 donors). **b,** TCF1 and LEF1 in expression in CD19.BBζ CAR T cells from one representative donor (*n* = 4 donors). **c,** IFNγ and IL-2 secretion from CD19.BBζ CAR T cells challenged with Nalm6 leukemia (*n* = 2–3 donors). **d,** Expression of memory- and exhaustion-associated markers on tNGFR-purified CD8^+^ CD19.BBζ CAR T cells before the first stimulation (left, pre-stim) and 7 days after the third stimulation (right, Stim 3). Graphs show mean ± s.e.m. of tNGFR-normalized mean fluorescence intensity from 2–3 donors. **e,** CD62L and IL7Rɑ in HA.28ζ CAR T cells. Contour plots show a representative donor from *n* = 5 donors. **f,** Cytotoxicity of HA.28ζ CAR T cells against 143B osteosarcoma cells (1:8 E:T, normalized to *t* = 0). Graphs show mean ± s.d. of 3 triplicate wells from one representative donor (*n* = 3 donors). **g,** Metabolic flux of CD19.28ζ CAR T cells measured by Seahorse XF analyzer (*n* = 2 donors). Plots show oxygen consumption rate (OCR) over time (left) from one representative donor, and ratio of OCR to extracellular acidification rate (ECAR, center) and spare respiratory capacity (SRC, right) from two donors. Graphs show the mean ± s.d. of each timepoint (left) or three representative timepoints (center, right) within each donor. **c,** 1-way ANOVA with Dunnett’s multiple comparison’s test**; d,** 2-way ANOVA or Mixed-effects model with Dunnett’s multiple comparison’s test; **g** unpaired two-sided student’s T test. *, *P* < 0.05; **, *P* < 0.01; ***, *P* < 0.001; ****, *P* < 0.0001.

**Figure 2:**
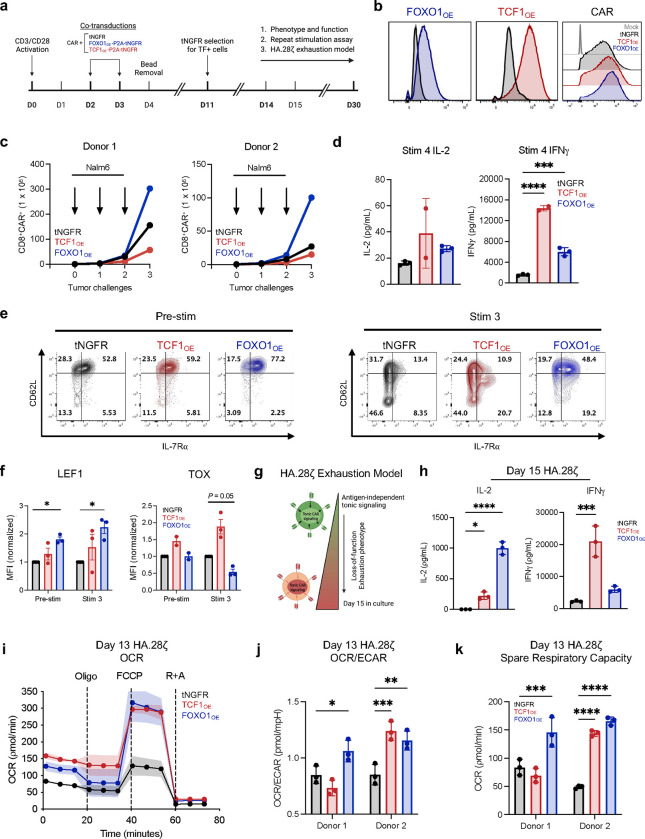
Overexpression of FOXO1 in CAR T cells promotes a memory phenotype, antitumor activity, and metabolic fitness during chronic stimulation. **a,** Schematic depicting engineering of truncated NGFR-only (tNGFR), TCF1/tNGFR- (TCF1_OE_), and FOXO1/tNGFR- (FOXO1_OE_) overexpressing CAR T cells and magnetic isolation of tNGFR positive cells for downstream analyses. **b,** Flow cytometric analysis confirming transcription factor overexpression in TCF1_OE_ (red) and FOXO1_OE_ (blue) compared to tNGFR (black) (left), and CD19.28ζ CAR expression across groups compared to untransduced cells (Mock, gray) (right). **c-f,** tNGFR-purified CD8^+^ CD19.BBζ CAR T cells overexpressing FOXO1 (blue), TCF1 (red), or tNGFR (black) were repeatedly challenged with Nalm6 at a 1:4 effector:target ratio. **c**, CD8^+^ T cell expansion measured 72h after each challenge (*n* = 2 donors). **d**, IL-2 and IFNγ secretion after the fourth tumor challenge (mean ± s.d. of 2–3 wells from 1 representative donor, *n* = 2 donors). **e,f,** Flow cytometric analysis of memory- and exhaustion-associated markers at baseline and 7 days after the third tumor challenge. Contour plots show a representative donor and bar graphs show mean ± s.e.m. of MFI normalized to tNGFR levels within each donor (*n* = 2–4 donors from 3 independent experiments). **g,** CAR T cell exhaustion model^15,24^ whereby T cells express a high-affinity GD2-targeting CAR (HA.28ζ) that promotes antigen-independent tonic CAR signaling for 15 days. **h,** Day 15 IL-2 and IFNγ secretion from HA.28ζ CAR T cells overexpressing tNGFR (black), TCF1 (red), or FOXO1 (blue) in response to 143B osteosarcoma cells. Plots show mean ± s.d. of 3 wells from 1 representative donor (*n* = 4 donors) **i-k,** Seahorse analysis was performed on tNGFR-purified HA.28ζ CAR T cells overexpressing tNGFR (black), TCF1 (red), or FOXO1 (blue) (*n* = 2 donors). **i,** Oxygen consumption rate (OCR) before and after treatment with oligomycin (Oligo), FCCP, and rotenone and antimycin (R+A). Plot shows mean ± s.d. from 11 technical replicates from one representative donor. **j,** Ratio of OCR to extracellular acidification rate (ECAR). **k,** Spare respiratory capacity. **j,k,** Bar graphs show mean ± s.d. of three representative time points within each donor. **d,** 1-way ANOVA with Dunnett multiple comparisons test. **f,h-k,** 2-way ANOVA with Dunnett’s multiple comparisons test or Mixed-effects model with Dunnett’s multiple comparisons test. *, *P* < 0.05; **, *P* < 0.01; ***, *P* < 0.001; ****, *P* < 0.0001.

Among the genes induced by FOXO1 is *TCF7*, which has been broadly implicated in memory programming and augmented functionality of human and mouse T cells^3,6,8,10,11,13,26–33^. We thus sought to determine whether overexpression of FOXO1 and/or TCF1 would enhance the function of human CAR T cells. Healthy donor T cells were retrovirally co-transduced with one virus expressing a CAR and a second virus expressing truncated NGFR (tNGFR) as a control or a bicistronic vector containing tNGFR and either TCF1 (TCF1_OE_) or FOXO1 (FOXO1_OE_) (Fig 2a,b). This approach enabled high TF overexpression and equivalent CAR expression across conditions (Fig. 2b). CD19.BBζ CAR T cells with or without TCF1 or FOXO1 overexpression (TCF1_OE_ or FOXO1_OE_), were magnetically selected for tNGFR-expressing cells and assessed for phenotype and function on day 14–16. FOXO1_OE_, but not TCF1_OE_, increased baseline expression of the memory-associated markers CD62L, IL-7Rα, and LEF1; FOXO1_OE_ also increased expression of endogenous TCF1^17,18^ (Extended Data Fig. 3a,b).

We next serially challenged CD19.BBζ CAR T cells with Nalm6 leukemia cells every 3 days. We observed that both TCF1_OE_ and FOXO1_OE_ cells displayed enhanced cytokine secretion after multiple stimulations compared to controls, but only FOXO1_OE_ augmented CD8 proliferation while preserving the expression of memory markers CD62L, IL-7Rα, and LEF1 while concomitantly suppressing TOX levels (Fig. 2c–f and Extended Data Fig. 3c,d). In contrast, TCF1_OE_ increased expression of TOX and CD39 relative to tNGFR controls, consistent with a more exhausted or effector-like phenotype (Fig. 2f and Extended Data Fig. 3d). We corroborated these results in cells expressing the tonic signaling HA.28ζ CAR, wherein both TCF1_OE_ and FOXO1_OE_ cells displayed enhanced function, but only FOXO1_OE_ induced a memory-like surface phenotype (Fig. 2g,h and Extended Data Fig. 3e,f).

Since memory T cells rely on oxidative phosphorylation (OXPHOS) metabolism relative to glycolysis, we used Seahorse assays to test whether FOXO1_OE_ and TCF1_OE_ induce similar memory-like metabolic profiles in non-tonic signaling CD19.28ζ or exhausted HA.28ζ CAR T cells. Indeed, T cells overexpressing either FOXO1 or TCF1 displayed similarly increased OXPHOS and superior metabolic fitness compared to tNGFR controls. The degree of FOXO1_OE_-mediated metabolic reprogramming was more dramatic in HA.28ζ CAR T cells (Fig. 2i–k) compared to those expressing CD19.28ζ (Extended Data Fig. 3g), consistent with the notion that FOXO1_OE_ counteracts the exhaustion program.

In summary, both TCF1_OE_ and FOXO1_OE_ enhanced CAR T cell function and metabolic fitness during settings of chronic stimulation. However, FOXO1_OE_ uniquely augmented CD8 proliferation and promoted a memory-like phenotype, whereas TCF1_OE_ enforced an exhaustion-like phenotype.

## FOXO1_OE_ CAR T cells exhibit a memory-like gene signature

**Extended Data Figure 4:**
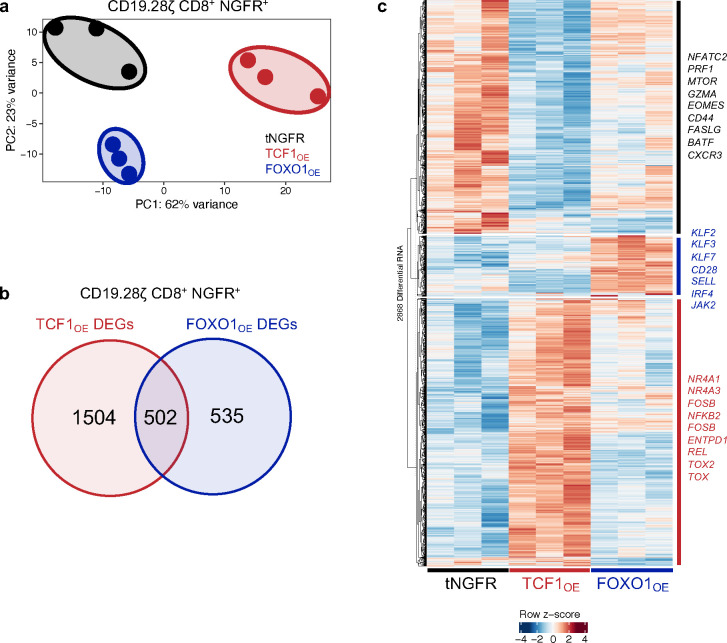
FOXO1 or TCF1 overexpression induces transcriptional reprogramming in CD19.28ζ CAR T cells. **a-c,** Bulk RNA sequencing analyses of day 15 tNGFR-purified CD8^+^ CD19.28ζ CAR T cells overexpressing tNGFR (black), TCF1 (red), or FOXO1 (blue) (*n* = 3 donors). **a,** Unbiased principal component analysis (PCA). **b,** Venn diagram showing the number of unique and shared differentially expressed genes (DEGs) in TCF1_OE_ and FOXO1_OE_ cells compared to tNGFR cells (adjusted *P* < 0.05 with log2(fold change) 0.5). **c,** Heatmap and hierarchical clustering of DEGs. Genes of interest are demarcated and colored based on the condition in which they’re upregulated.

**Figure 3.**
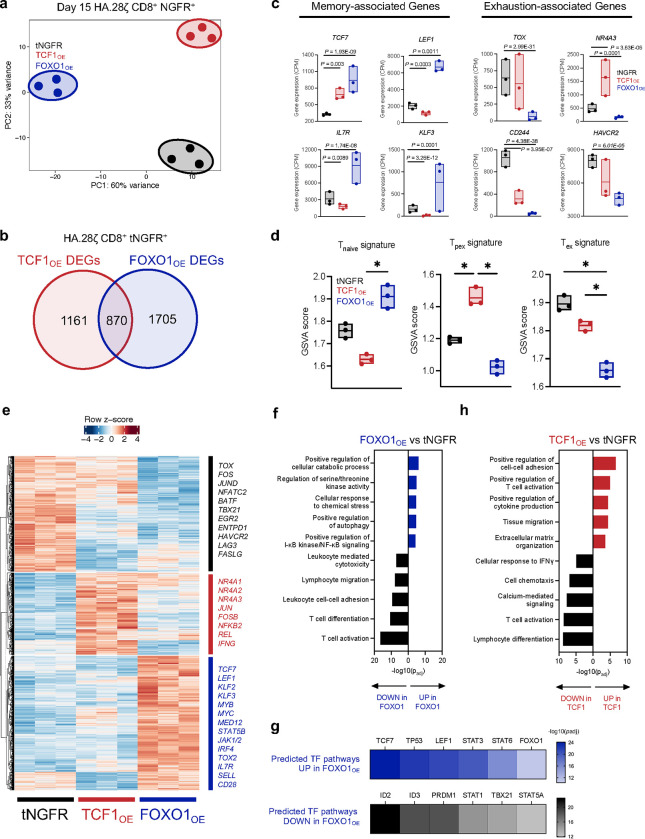
Overexpression of FOXO1, but not TCF1, induces a memory-like transcriptional program. **a-h,** Bulk RNA-sequencing analyses of day 15 tNGFR-purified CD8^+^ HA.28ζ CAR T cells overexpressing tNGFR (black), TCF1 (red), or FOXO1 (blue) (*n* = 3 donors). **a,** Unbiased principal component analysis (PCA). **b,** Venn diagram showing the number of unique and shared differentially expressed genes (DEGs) in TCF1_OE_ and FOXO1_OE_ cells compared to tNGFR cells (adjusted *P* < 0.05 with log2(fold change) 0.5). **c,** Expression of memory- (left) and exhaustion-associated (right) genes. Center line represents the mean counts per million of 3 donors. **d,** Gene set variation analysis (GSVA) using naive, progenitor exhausted, and exhausted T cell signatures from Andreatta et al^66^. **e,** Heatmap and hierarchical clustering of DEGs. Genes of interest are demarcated and colored based on the condition in which they’re upregulated. **f,** Gene ontology (GO) term analyses showing curated lists of top up- and downregulated processes in FOXO1_OE_ cells (left) TCF1_OE_ cells (right) versus tNGFR controls. **g,** QIAGEN Ingenuity Pathway Analysis (IPA) of upregulated and downregulated transcription factor pathways in FOXO1_OE_ cells versus tNGFR controls. **c**, Paired analyses using DESeq2. **d**, One-way ANOVA with Tukey’s multiple comparisons test. *, *P* < 0.05.

We hypothesized that FOXO1 and TCF1 induce disparate gene expression programs since overexpression of each endowed CAR T cells with distinct cell surface phenotypes and functionality (Fig. 2). Therefore, we performed bulk RNA sequencing on purified CD8^+^ FOXO1_OE_ and TCF1_OE_ T cells expressing either HA.28ζ or CD19.28ζ CARs to model settings with or without tonic signaling, respectively. Principal component analyses showed that FOXO1_OE_ and TCF1_OE_ CAR T cells cluster separately from tNGFR along PC1 and PC2 (Fig. 3a and Extended Data Fig. 4a) and displayed a greater number of unique differentially expressed genes (DEGs vs tNGFR, FDR<0.05) compared to those that were shared (Fig. 3b, Extended Data Fig. 4b), confirming that FOXO1_OE_ and TCF1_OE_ promote divergent gene expression programs. Of note, FOXO1_OE_ exhibited a greater number of DEGs in tonic signaling HA.28ζ CAR T cells compared to those expressing CD19.28ζ (Fig. 3b and Extended Data Fig. 4b), indicating more dramatic transcriptional reprogramming by FOXO1 in settings of chronic stimulation (Fig. 2).

Consistent with protein data, HA.28ζ FOXO1_OE_ cells upregulated genes associated with memory (*SELL, IL7R, LEF1, TCF7, KLF3*) and downregulated exhaustion-associated genes (*TOX, HAVCR2, ENTPD1,* and *CD244*) (Fig. 3c–e)*.* Gene set variation analyses (GSVA) corroborated these data, wherein FOXO1_OE_ promoted a naive-like and less terminally exhausted gene signature (Fig 3d). Similar results were obtained in CD19.28ζ CAR T cells (Extended Data Fig 4c). Gene ontology (GO) of upregulated FOXO1 genes identified STAT3^34^, autophagy^35^, cellular stress response, and most notably, cellular catabolism (Fig. 3f), consistent with FOXO1_OE_-induced metabolic reprogramming (Fig. 2i–k). Ingenuity Pathway Analysis (IPA) identified that memory and naive-associated TF gene expression networks were enriched (*TCF7*, *LEF1*, *STAT6*) while effector TF expression networks were diminished (*ID2, PRDM1, TBX21*) in FOXO1_OE_ cells compared to tNGFR controls, further underscoring the degree of global memory reprogramming induced by FOXO1_OE_ (Figure 3g). In contrast, TCF1_OE_ cells exhibited high expression of exhaustion-associated NR4A and AP-1 family TFs (Fig. 3c,e), a T_PEX_-like gene signature (Fig. 3d), and were enriched in effector gene expression pathways (ex. cell-cell adhesion, T cell activation, cytokine production) (Fig. 3h). Together, these data demonstrate that FOXO1_OE_ induces memory and naive-like gene expression programs during chronic stimulation, whereas TCF1_OE_ promotes a progenitor exhaustion-like or effector program, consistent with the role identified for TCF1 in chronic infection and cancer^26,27,36,37^.

## FOXO1 and TCF1 overexpression induce chromatin remodeling at their putative DNA binding motifs

**Extended Data Figure 5:**
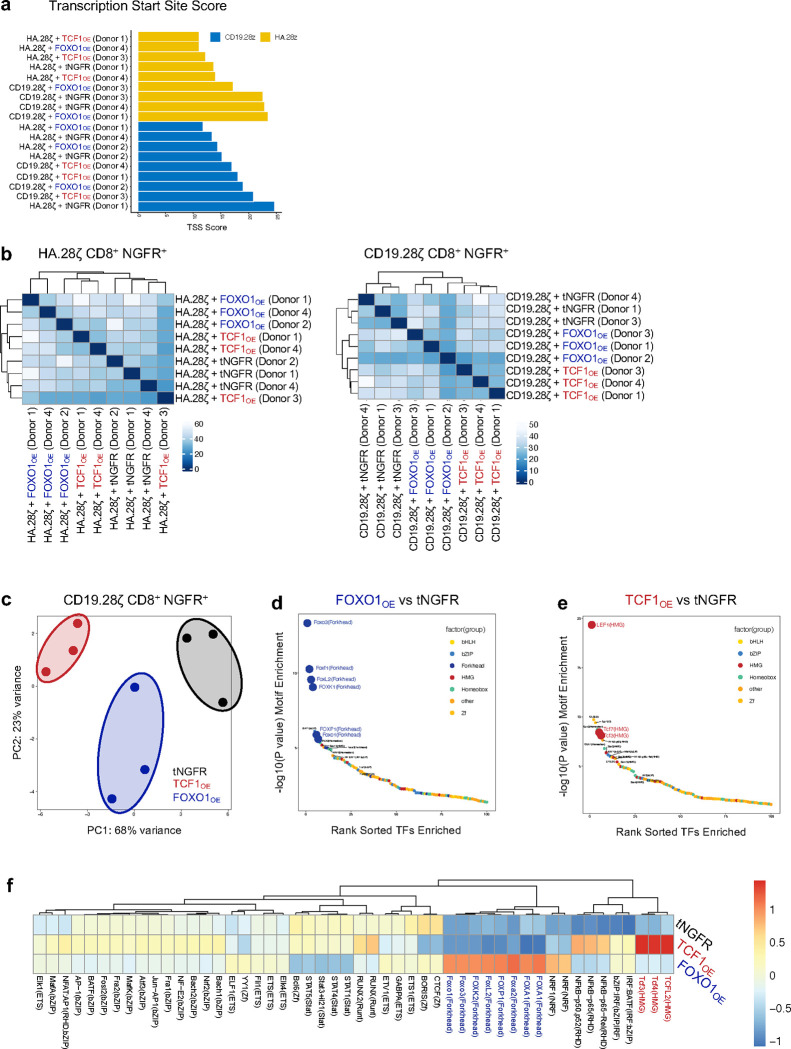
FOXO1 and TCF1 overexpression induce chromatin remodeling in HA.28ζ and CD19.28ζ CAR T cells. **a,** Transcriptional start site (TSS) enrichment scores for all samples. **b,** Pearson correlation and hierarchical clustering of ATAC-sequencing data. **c-f,** Bulk ATAC-seq analyses of day 15 tNGFR-purified, CD8+ CD19.28ζ CAR T cells overexpressing tNGFR (black), TCF1 (red) or FOXO1 (blue) (*n* = 3 independent donors). **c,** Principal component analysis (PCA). **d,e,** Rank ordered plot of differentially accessible transcription factor binding motifs in FOXO1_OE_ cells (**d**) and TCF1_OE_ cells (**e**) versus tNGFR controls. Transcription factor families are annotated by color. **f,** Heatmap and hierarchical clustering of mean differential motif accessibility. Scale shows normalized z-scores for each motif.

**Figure 4.**
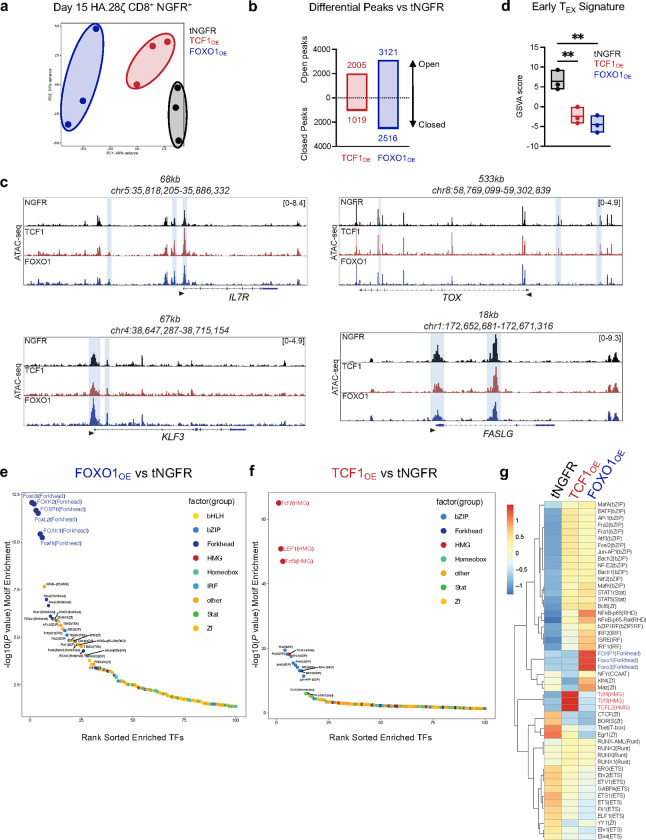
FOXO1 and TCF1 overexpression induce chromatin remodeling at their putative DNA-binding motifs. **a-g,** Bulk ATAC-sequencing analyses of day 15 tNGFR-purified CD8^+^ HA.28ζ CAR T cells overexpressing tNGFR (black), TCF1 (red), or FOXO1 (blue) (*n =* 3 donors). a, Principal component analysis (PCA). b, Differential open and closed peaks compared to tNGFR controls c, Chromatin accessibility tracks at *IL7R, TOX, KLF3,* and *FASLG* loci for a representative donor. d, Enrichment of an early exhausted T cell chromatin accessibility signature based on ATAC-seq data from Satpathy et al.^9^ Center line represents the mean of 3 donors. e,f, Rank ordered plot of differentially accessible transcription factor binding motifs in FOXO1_OE_ cells (e) and TCF1_OE_ cells (f) versus tNGFR controls. Transcription factor families are annotated by color. g, Heatmap and hierarchical clustering of mean differential motif accessibility. Scale shows normalized z-scores for each motif.

Recent work showed that overexpression of the AP-1 family TF, c-Jun, induced transcriptional reprogramming and promoted exhaustion resistance in human T cells, but did not alter the epigenome^24^. In contrast, FOXO1 and TCF1 are considered pioneer factors due to their ability to directly bind and open condensed chromatin and recruit chromatin remodeling machinery^38–40^. To test whether TCF1_OE_ and/or FOXO1_OE_ induce epigenetic remodeling, we performed bulk ATAC-seq on CD8^+^ NGFR+ CAR T cells expressing either CD19.28ζ or HA.28ζ CARs (Extended Data Fig. 5a,b). Principal component analyses confirmed that OE of either TF promoted global changes to chromatin accessibility compared to tNGFR controls (Fig. 4a and Extended Data Fig. 5c). This effect was most evident with FOXO1_OE_ in HA.28ζ CAR T cells, which clustered separately from tNGFR and TCF1 groups along PC1 (Fig. 4a). and displayed more differentially accessible peaks (~5600, *P* < 0.05) compared to TCF1_OE_ cells (~3000) (Fig. 4b). The majority of differentially accessible genes were open compared to controls, consistent with FOXO1’s ability to perturb core histone:DNA contacts^41^.

HA.28ζ FOXO1_OE_ cells displayed increased accessibility at FOXO1 target gene loci (*IL7R* and *KLF3*), reduced accessibility of exhaustion-associated loci (*TOX* and *FASLG*) (Fig. 4c), and a decreased exhaustion-like epigenetic signature compared to tNGFR cells (Fig. 4d), consistent with the transcriptomic data (Fig. 3). Of note, forkhead box and HMG-box family TF DNA-binding motifs were the top-ranked differentially accessible motifs in FOXO1_OE_ and TCF1_OE_ cells, respectively, in both CD19.28ζ and HA.28ζ T cells, supporting a model in which overexpressed FOXO1 and TCF1 function as pioneer TFs that induce local chromatin remodeling (Fig. 4e,f and Extended Data Fig. 5d,e). FOXO1_OE_ cells (and to a lesser extent TCF1_OE_ cells) also exhibited decreased accessibility at motifs bound by TFs that promote T cell differentiation (ex. *TBX21*, *EOMES*, *EGR,* and *FLI1*), but paradoxically, exhibited an increase at those which are associated with effector function (ex. *b-ZIP* and *NFKB-p65*) (Figure 4e–g, Extended Data Fig. 4f). These data demonstrate that FOXO1_OE_ induces a unique epigenetic state that supports effector function while maintaining memory programming.

## Wild-type FOXO1, but not a nuclear-restricted variant, augments CAR T persistence and antitumor function in vivo

**Extended Data Figure 6:**
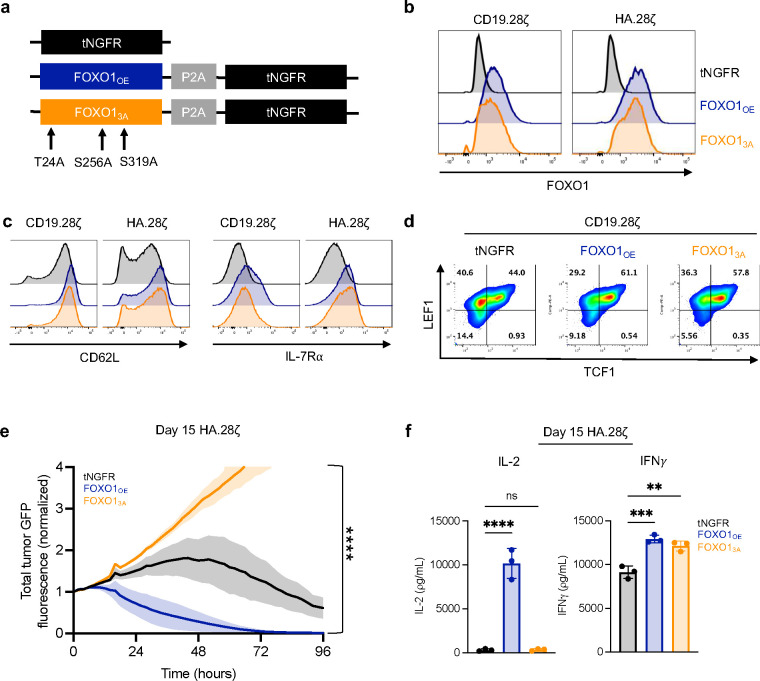
Nuclear-restricted FOXO1 promotes a memory-like phenotype but demonstrates impaired effector function. **a,** Schematic showing a mutated variant of FOXO1 that contains three amino acid substitutions (T24A, S256A, and S319A) which restrict nuclear export (FOXO1_3A_, orange). **b,** FOXO1 expression in CD19.28ζ and HA.28ζ CAR T cells from one representative donor (*n* = 5 donors). **c,** CD62L and IL7Ra expression in CD19.28ζ and HA.28ζ CAR T cells from one representative donor (*n* = 3 donors). **d,** TCF1 and LEF1 expression in CD19.28ζ CAR T cells from one representative donor (*n* = 3 donors). **e,** Cytotoxicity of HA.28ζ CAR T cells against Nalm6 leukemia (1:1 E:T, normalized to *t* = 0). Graph shows mean ± s.d. from one representative donor (*n* = 3 donors). **e,** 2-way ANOVA with Bonferroni’s multiple comparisons test. **f,** 1-way ANOVA with Dunnett’s multiple comparison’s test. **, *P* < 0.01; ***, *P* < 0.001; ****, *P* < 0.0001.

Since FOXO1_OE_ was effective at enhancing CAR T function (Figs. 2,3,4), we hypothesized that further increasing FOXO1 activity could endow CAR T cells with a more stable memory phenotype. Prior work in mice showed that a nuclear-restricted variant of FOXO1 (FOXO1_3A_), which is insensitive to Akt-mediated nuclear export, promotes T cell persistence during chronic infection^20^. Therefore, we generated and tested a humanized version of FOXO1_3A_ in CAR T cell models of chronic stimulation (Extended Data Fig. 6a,b). FOXO1_3A_ increased surface expression of FOXO1 target genes (Extended Data Fig. 6c,d); however, unexpectedly, FOXO1_3A_ cells displayed reduced *in vitro* cytokine secretion and cytotoxicity compared to FOXO1_OE_ (Extended Data Fig. 6 e,f). These observations raised the prospect that excessive FOXO1 activity might promote a stable memory phenotype while concomitantly opposing effector function^42^.

**Extended Data Figure 7:**
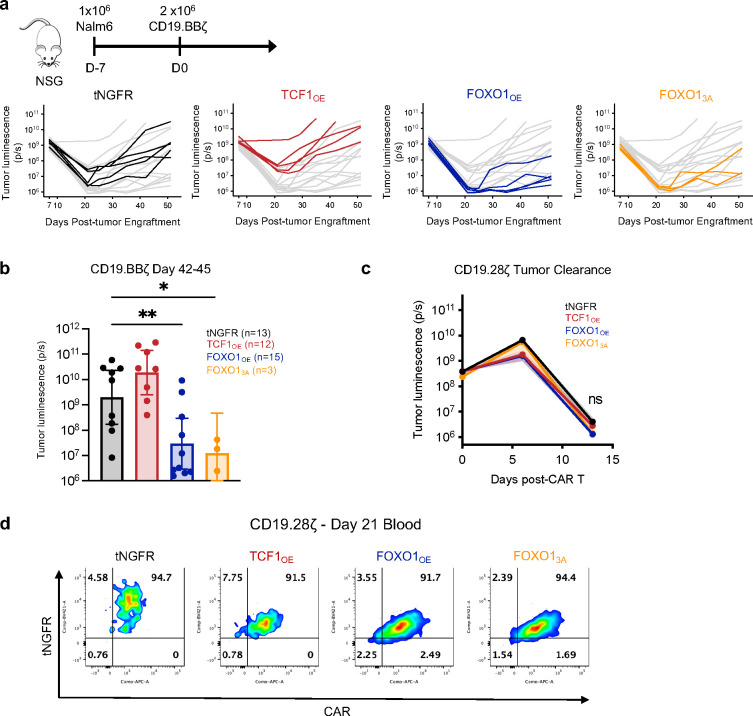
FOXO1_OE_ CAR T cells demonstrate enhanced anti-tumor activity *in vivo.* **a,b,** A curative dose of 2×10^6^ tNGFR-purified CD19.BBζ CAR T cells overexpressing tNGFR (black), TCF1 (red), FOXO1 (blue), or FOXO1_3A_ (orange) were infused into Nalm6 leukemia-bearing mice 7 days post-engraftment. **a,** Experimental schematic (top) and tumor bioluminescence of multiple timepoints from 1 representative donor (bottom) (n = 3–5 mice per group). **b,** Combined tumor bioluminescence data from 2 donors at day 42–45 (*n* = 3–10 mice per group; 1 donor for FOXO1_3A_). **c,** Tumor bioluminescence data for Nalm6-bearing mice injected with a curative dose of 2×10^6^ CD19.28ζ CAR T cells outlined in Figure 5b. **d,** CD19.28ζ CAR and tNGFR expression on circulating human CD45+ cells on day 21 post-CAR T infusion. These data show one representative mouse from each group and corresponds to Figures 5b–d. **b,** Mann-Whitney test. **c,** 2-way ANOVA with Dunnett’s multiple comparisons test. *, *P* < 0.05; **, *P* < 0.01; ns, *P* > 0.05.

**Figure 5.**
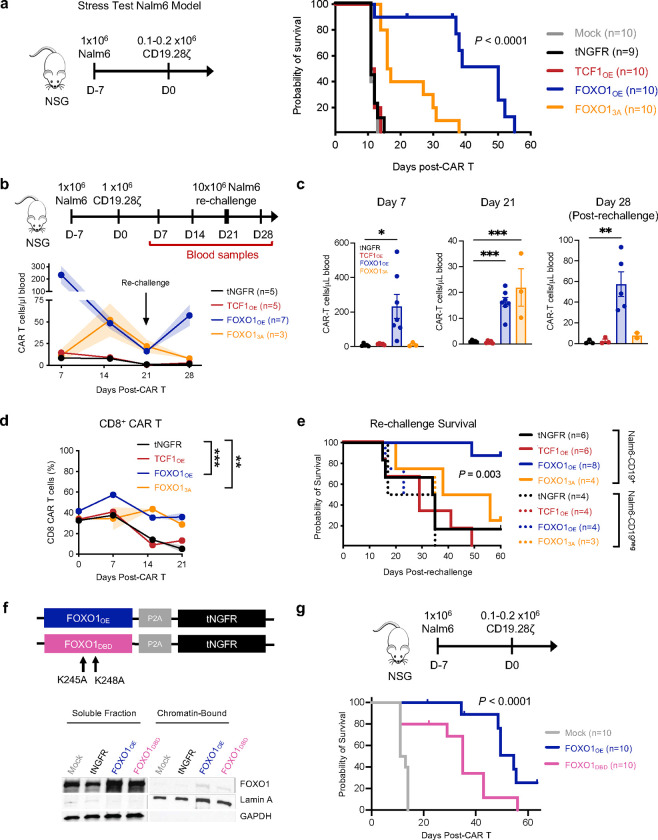
FOXO1 overexpression enhances CAR T cell expansion, persistence, and antitumor activity in leukemia xenograft models. **a,** A subcurative dose of 0.1–0.2×10^6^ tNGFR-purified CD19.28ζ CAR T cells were infused into Nalm6 leukemia-bearing mice 7 days post-engraftment. Stress test Nalm6 model schematic (left) and survival curve (right) are shown (*P* < 0.0001 log-rank Mantel-Cox test). Data are from 2 donors (*n* = 4–5 mice per condition). **b-d,** A curative dose of 1×10^6^ tNGFR-purified CD19.28ζ CAR T cells overexpressing tNGFR (black), TCF1 (red), FOXO1 (blue), or FOXO1_3A_ (orange) were infused into Nalm6 leukemia-bearing mice 7 days post-engraftment. Mice were rechallenged with 10×10^6^ Nalm6 leukemia cells on day 21 post-CAR T cell infusion (**b**, top). **b,c,d** Quantification of human CD45+ CAR T cells in peripheral blood harvested on days 7, 14, 21, and 28 (**b,c**) and percent CD8+ CAR T cells (**d**) by flow cytometry. Plots show mean ± s.e.m. of 3–7 mice per group from 1 representative donor (*n* = 2 donors). **e,** Kaplan-Meier curve depicting survival of mice after rechallenged with CD19+ or CD19- Nalm6 leukemia cells (*P* = 0.003, log-rank Mantel-Cox test). Combined data from two donors are shown (*n* = 3–8 mice per group). **f,** Schematic depicting construct design and amino acid substitutions to generate human FOXO1_DBD_ (pink, top) and Western blots of indicated proteins in soluble and chromatin-bound fractions isolated from day 8 post-activation tNGFR-purified CD19.28ζ CAR T cells (bottom). **g,** Schematic of CD19.28ζ stress test Nalm6 model (as shown in **a**) comparing mock and FOXO1-WT- or FOXO1-DBD_mut_-overexpressing CAR T cells (above) and survival curve (below) (*P* < 0.0001, log-rank Mantel-Cox test). Combined data from two donors are shown (*n* = 10 mice per group, data from 1 donor is also included in **a**).

To assess functionality in a longer-term model where memory programming may be important for sustained responses, we performed a stress test xenograft model in which Nalm6 leukemia-bearing mice were infused with a subtherapeutic dose of TF-engineered CD19.28ζ (Fig. 5a) or CD19.BBζ CAR T cells (Extended Data Fig. 7a). Mice infused with FOXO1_OE_ CAR T cells exhibited augmented tumor control compared to those infused with control tNGFR cells, whereas mice infused with TCF1_OE_ cells showed no benefit (Fig. 5a and Extended Data Fig. 7a,b). Similar results were obtained in a curative Nalm6 model (Extended Data Fig. 7c), wherein FOXO1_OE_ CD19.28ζ CAR T cells displayed a profound advantage in overall expansion, persistence, and CD8^+^ frequency compared to TCF1_OE_ and tNGFR controls (Fig. 5b–d). FOXO1_3A_ cells exhibited augmented antitumor function compared to tNGFR controls but showed delayed expansion and reduced tumor control compared to FOXO1_OE_, consistent with the notion that FOXO1_3A_ partially opposes effector function (Fig. 5b–d). To assess recall response to secondary antigen challenge, a hallmark feature of memory T cells^43^, we rechallenged nearly-cured mice with a high dose of 1×10^7^ Nalm6 cells on day 21 post-CAR-T-infusion (Fig. 7b and Extended Data Fig. 7d). Only FOXO1_OE_ cells re-expanded after re-challenge and promoted a survival advantage (Fig. 5b,c,e), demonstrating that FOXO1_OE_ endows CAR T cells with superior effector- and memory-like functionality compared to tNGFR controls, TCF1_OE_ or FOXO1_3A_.

**Figure 6.**
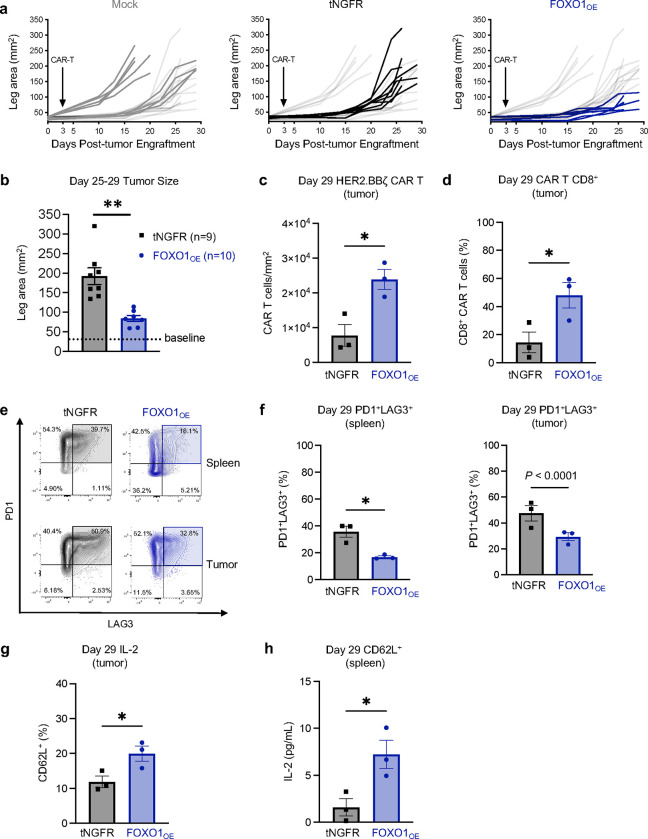
FOXO1_OE_ CAR T cells exhibit improved antitumor activity in a solid tumor xenograft model. 5×10^6^ Her2.BBζ CAR T cells were infused into 143B osteosarcoma-bearing mice 3 days post-engraftment. **a-b** Tumor growth of individual mice treated with mock (grey), tNGFR (black), or FOXO1_OE_ (blue) cells. Combined temporal data from 2 donors is shown in (**a**) (*n* = 8–9 mice per group) and at a selected timepoint between day 25–29 (*n* = 7–8 mice per group). **c-h,** Tumors and spleens were harvested and processed for phenotypic and functional assays on day 29 post-engraftment (1 donor, *n* = 3 mice per group). **c,** Total tumor-infiltrating CAR T cells. **d,** Percent CD8^+^ tumor-infiltrating CAR T cells. **e,f,** Exhaustion marker expression from splenic and tumor-infiltrating CAR T cells. Contour plots show 1 representative mouse and bar graphs show mean ± s.e.m. of 3 mice per group. **g,** CD62L expression from splenic CAR T cells **h,** IL-2 secretion after *ex vivo* stimulation with cultured 143B. Data shows the mean ± s.e.m. of 3 mice. **b-d, f-h**, unpaired two-sided student’s T test. *, *P* < 0.05; **, *P* < 0.01.

Importantly, mice infused with CD19.28ζ cells expressing a DNA-binding domain mutant variant of FOXO1 (FOXO1_DBD_), which had a modest reduction in DNA binding, exhibited reduced survival in a Nalm6 leukemia stress test model compared to those infused with FOXO1_OE_ cells, indicating that augmented antitumor activity endowed by FOXO1_OE_ is dependent on DNA-binding (Fig. 5f,g). To determine whether FOXO1 was also capable of augmenting CAR T activity against solid tumors, we infused tNGFR control or FOXO1_OE_ HER2.BBζ CAR T cells into 143B osteosarcoma-bearing NSG mice. Consistent with leukemia models, FOXO1_OE_ CAR T cells displayed enhanced antitumor activity and persistence (Fig. 6a–c), increased CD8^+^ persistence (Fig. 6d), diminished inhibitory receptor expression and increased CD62L (Fig. 6e–g), and augmented cytokine secretion following *ex vivo* stimulation of tumor-infiltrating CAR T cells (Fig. 6h).

Taken together, these data demonstrate that FOXO1_OE_ augments CAR T expansion, persistence, and tumor control *in vivo*, whereas TCF1_OE_ provides no measurable benefit. FOXO1_OE_-mediated enhancements are dependent on DNA binding and nuclear export, suggesting that tuning or signal regulation mediated by nuclear shuttling is important for effective FOXO1-mediated memory programming.

## FOXO1 activity correlates with response to T cell-based immunotherapies

**Extended Data Figure 8:**
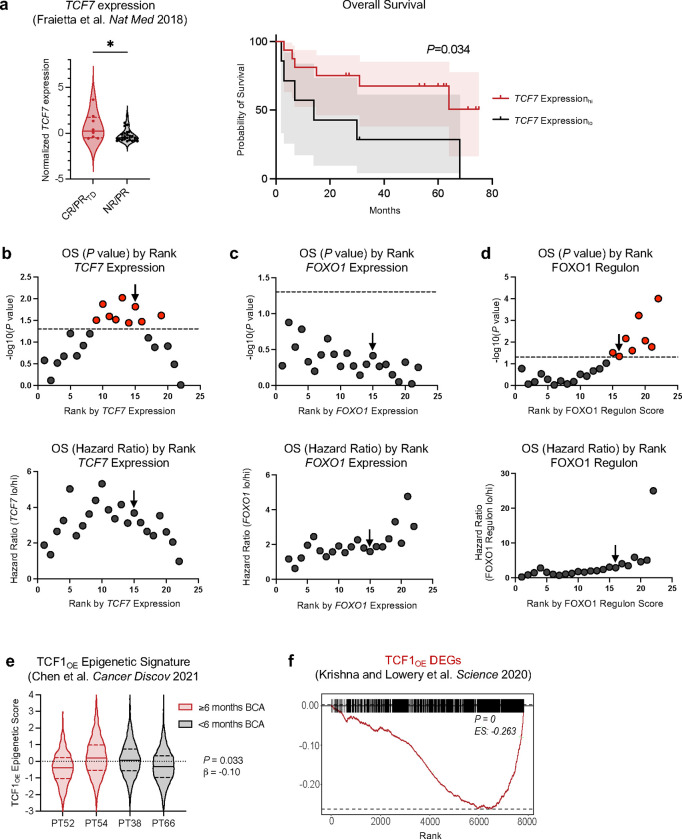
Endogenous *TCF7* transcript and FOXO1 regulon, but not TCF_OE_ transcriptional or epigenetic signatures, predict CAR T and TIL responses in patients. **a,** Single-sample gene set enrichment analyses (GSEA) were performed on RNA-sequencing data generated from *ex vivo* CAR-stimulated patient CTL019 T cells published in Fraietta et al. 2018 (cite). Enrichment score stratification points for patient survival analyses were determined using previously published methods (Jung et al 2023). *TCF7* transcript correlates with response to CAR T (left) and overall survival (right). **b-d**, *P* values (top) and hazard ratios (bottom) of different stratification points in relation to overall survival (OS) of *TCF7* expression (**b**), *FOXO1* expression (**c**), and FOXO1 regulon (**d**). Dotted lines are drawn at *P* < 0.05, and black arrows indicate the stratification points used. **e**, Epigenetic signatures derived from differentially accessible peaks *(P <* 0.05) in CD19.28ζ TCF1_OE_ CD8^+^ cells vs. tNGFR controls (Extended Data Fig. 5) were applied to scATAC-seq data generated from B-ALL apheresed patient T cells published in Chen et al^6^. The TCF1_OE_-derived epigenetic signature was not associated with patients with durable CAR T persistence (≥ 6 months B cell aplasia, BCA; Patient 52, *n* = 616 cells; Patient 54, *n* = 2959 cells) compared to those with short persistence (< 6 months BCA, Patient 38, *n* = 2093 cells; Patient 66, *n* = 2355 cells). **f**, GSEA analyses were performed on DEGs from CD39^−^CD69^−^ patient TIL that correlated with responses in adult melanoma (Krishna and Lowery et al). HA.28ζ TCF1_OE_ CD8^+^ DEGs (Fig. 3) were significantly de-enriched in CD39^−^CD69^−^ TIL. **a**, Mann-Whitney test (left), Mantel-Cox test (right); **e**, Wald test of a linear regression model.

**Figure 7.**
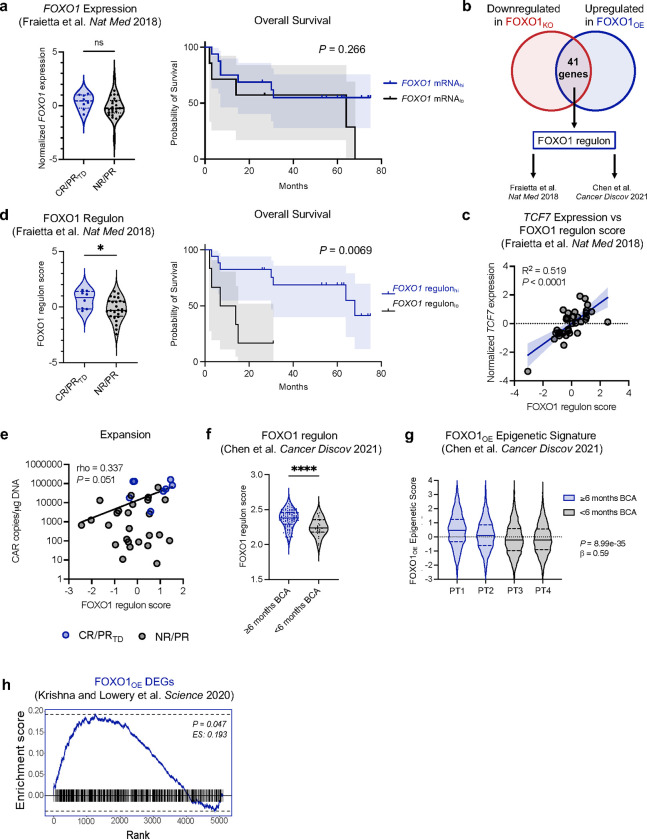
FOXO1 activity correlates with clinical response to CAR T and TIL therapy. **a-d**, Single-sample gene set enrichment analyses (GSEA) were performed on RNA-sequencing data generated from *ex vivo* CAR-stimulated patient CTL019 T cells published in Fraietta et al.^3^ (complete responder, CR, *n* = 5; partial responder with transformed disease, PR_TD_, *n* = 3; partial responder, PR, *n* = 5; non-responder, NR, *n* = 21). **a**, *FOXO1* transcript did not correlate with response to CAR T (left) or overall survival (right). **b**, An empiric gene signature representing the FOXO1 regulon (Table 1) was then applied to the same data set. **c**, Simple linear regression showing the correlation between *TCF7* expression and FOXO1 regulon score. Dots shown are individual CTL019 patient samples and shaded area indicates 95% confidence intervals. **d**, FOXO1 regulon scores significantly correlated with response (left), overall survival (right), and **e**, trended with CAR T peak expansion. **f**, Single-sample GSEA analyses on RNA-sequencing data from pediatric B cell acute lymphocytic leukemia (B-ALL) patient-apheresed effector T cells which were subsequently manufactured with CD19.BBζ and infused into patients^6^. The FOXO1 regulon was enriched in patients who exhibited durable CAR T persistence (≥6 months B cell aplasia, BCA; *n* = 33 patients) compared to those with short persistence (< 6 months BCA, *n* = 27 patients). **g**, Epigenetic signatures derived from differentially accessible peaks (*P* < 0.05) in CD8+ CD19.28ζ FOXO1_OE_ and TCF1_OE_ cells (Fig. 3 and Extended Data Fig. 5) were applied to scATAC-seq data generated from B-ALL apheresed patient T cells published in Chen et al.^6^ The FOXO1_OE_-derived epigenetic signature was significantly enriched in cells from two patients with durable CAR T persistence (≥6 months BCA, Patient 52, *n* = 616 cells; Patient 54, *n* = 2959 cells) compared to those with short persistence (< 6 months BCA, Patient 38, *n* = 2093 cells; Patient 66, *n* = 2355 cells). **h**, GSEA analyses were performed with CD8^+^ HA.28ζ FOXO1_OE_ differentially expressed genes (DEGs, Fig. 3) and DEGs from CD39^−^CD69^−^ patient TIL that correlated with TIL therapy responses in adult melanoma from Krishna and Lowery et al.^8^ **a**, Mann-Whitney test (left); Mantel-Cox test (right); **d,f,** Mann-Whitney test; **e**, Spearman correlation; **g**, Wald test of a linear regression model.

FOXO1 target genes, including *TCF7*, and related pathways (ex. IL-6/STAT3) were enriched in pre-infusion CAR T cells that mediated clinical responses in patients^3,6,44^ (Extended Data Fig. 8a,b), raising the prospect that endogenous FOXO1 activity might be gating for potent antitumor activity in clinical CAR T cell products. Paradoxically, however, *FOXO1* transcript levels in manufactured CD19.BBζ CAR T cells were not associated with response to therapy or survival in adult chronic lymphocytic leukemia (CLL) patients (Fig. 7a, Extended Data Fig. 8c). Since FOXO1 is primarily regulated at the post-translational level in an Akt-dependent manner rather than through dynamic changes in gene expression^45^, we hypothesized that FOXO1 activity could be better approximated by the aggregate expression of FOXO1 target genes. Using an unbiased approach, we identified a FOXO1 “regulon” consisting of overlapping DEGs that were downregulated in FOXO1_KO_ cells and upregulated in FOXO1_OE_ cells (Fig. 7b). We identified a list of 41 putative FOXO1 target genes, which included previously described genes, such as *SELL* and *KLF3,* but was largely comprised of genes not previously associated with memory programming (Table 1). *TCF7* did not reach statistical significance in FOXO1_KO_ experiments and was therefore not included in the FOXO1 regulon; however, regulon score significantly correlated with *TCF7* transcript in patient CAR T cells, suggesting that the regulon is an accurate readout for FOXO1 transcriptional activity (Fig. 7c). The FOXO1 regulon was significantly enriched in pre-infusion CAR T cells from adult chronic lymphocytic leukemia (CLL) patients^3^ who exhibited complete responses or partial responses with transformed disease, and was associated with CAR T expansion and overall survival (Fig. 7d,e and Extended Data Fig. 8d). The FOXO1 regulon was also enriched in premanufactured effector T cells from pediatric B cell acute lymphoblastic leukemia (B-ALL) patients with durable CAR T persistence^6^ (≥ 6 months B cell aplasia, Fig. 7f).

Since both FOXO1 and TCF1 mediate chromatin remodeling^38,46–50^ (Fig. 4), we next utilized epigenetic signatures derived from TF-overexpressing CAR T cells to interrogate single-cell ATAC-seq data from pediatric B-ALL patient premanufactured T cells^6^. Consistent with FOXO1 regulon data, the FOXO1_OE_ epigenetic signature was significantly enriched in patient T cells that were associated with durable persistence, whereas the TCF1_OE_ signature was not (Fig. 7g and Extended Data Fig. 8e). Finally, FOXO1_OE_ DEGs were highly enriched in a CD39^−^CD69^−^ subset of TILs that were highly predictive of response in melanoma patients^8^, whereas TCF1_OE_ DEGs were de-enriched (Fig 7h, Extended Data Fig. 8f).

Collectively, these data demonstrate that FOXO1-driven transcriptional and epigenetic programs are associated with engineered and non-engineered T cells that expand, persist, and promote clinical responses in cancer patients and that these properties can be endowed upon human T cells by overexpression of FOXO1. Further, these results are consistent with a model whereby the high level of correlation between T cell memory phenotype and function and *TCF7* transcript reflects FOXO1 transcriptional and epigenetic programming.

## DISCUSSION

Several studies have identified genes and pathways associated with improved CAR T response in patients^3–7^, but a mechanistic understanding of the transcription factors which promote these pathways and bioengineering approaches to leverage them are lacking. In this study, we tested the hypothesis that overexpression of memory-associated TFs could reprogram CAR T cells with enhanced persistence and antitumor activity. We focused our efforts on TCF1, a TF which defines T cell populations with enhanced stemness, memory properties, and augmented capacity to respond to immune checkpoint blockade^3,6,8,10,11,13,26–33^. We also analyzed the effects of FOXO1 based upon studies implicating this TF in memory programming^17–20,23,34,42,51–59^ and our previous work wherein exhaustion reversal and memory programming was associated with enhanced chromatin accessibility at FOXO1 binding motifs^15^. FOXO1 overexpression induced memory gene expression programs and chromatin remodeling, mitigated exhaustion, and substantially improved persistence and antitumor function in four distinct xenograft models. Additionally, its effect was independent of CAR binder, co-stimulatory domain, and tumor type, highlighting the broad application of this pro-memory program across CAR T products. In contrast, TCF1 overexpression enforced T_PEX_-like programs and failed to augment CAR T responses *in vivo*.

There is a vast body of literature describing the role of FOXO1 in promoting T cell memory and persistence in mice^17–20,23,34,42,51–59^; however, FOXO1 biology in human T cells remains poorly understood. Our study is the first to demonstrate that endogenous FOXO1 is required for promoting antitumor function in human engineered T cells, results consistent with findings in murine models of acute and chronic infection^19,20,23^. We further demonstrated that FOXO1 restrains exhaustion in human T cells, since quiescent FOXO1_KO_ CAR T cells exhibit increased expression of surface markers and genes associated with terminal differentiation and exhaustion. Notably, endogenous FOXO1 activity in pre-infusion patient CAR T cells or TILs strongly correlates with clinical responses, underscoring the importance of endogenous FOXO1 in T cell-based cancer immunotherapies. Additional work is needed to determine the exact level of activity and the specific gene expression programs induced by endogenous FOXO1 during CAR T manufacturing, and whether endogenous FOXO1 is relevant in other therapeutic modalities, such as immune checkpoint blockade.

FOXO1 overexpression promotes memory-associated gene expression programs and increases chromatin accessibility at forkhead box family TF motifs, consistent with its function as a pioneer factor^40,60^. Experiments utilizing a FOXO1 DNA-binding mutant suggest that both transcriptional and epigenetic changes induced by FOXO1_OE_-binding are required for enhanced antitumor function. Paradoxically, further increasing FOXO1 activity by overexpressing a nuclear-restricted variant (FOXO1_3A_) attenuates CAR T antitumor function, supporting the notion that optimal FOXO1 activity involves intermittent and/or context-dependent regulation. Indeed, others have demonstrated that transient expression of FOXO1_3A_ can induce memory reprogramming in human CAR T cells^21,61,62^, and FOXO1_3A_ expression depletes mouse tumor-infiltrating regulatory T cells to enhance antitumor immunity^63^. Additional studies are needed to understand how exogenous FOXO1 levels, expression kinetics, and DNA binding activity affect CAR T cell state and function, and whether FOXO1_OE_ differentially affects distinct T cell subsets.

We also present the unexpected finding that TCF1 overexpression fails to enforce memory gene expression programs or enhance antitumor activity in vivo, which contradicts reports in mice^28,29^. Instead, TCF1_OE_ in tonic signaling CAR T cells upregulates markers associated with exhaustion, such as NR4A family TFs and *ENTPD1*, consistent with a recent study which showed that endogenous TCF1 repressed effector T cell differentiation and promoted exhaustion during murine chronic infection^37^. An additional study found that T cell receptor- (TCR) engineered T cells with TCF1 *TRAC* knock-in were associated with diminished *IL2* and *TNF* transcript *in vivo*^64^. Thus, our results raise the prospect that constitutive TCF1 overexpression skews human engineered T cells towards a more exhausted or T_PEX_-like cell state and/or that *TCF7*-expressing T_PEX_, which reside in lymph nodes and are critical for checkpoint blockade efficacy^26,27,36^, do not play a substantial role in CAR T responses.

An alternative interpretation posits that FOXO1, rather than TCF1, is primarily responsible for endowing a stem-like or progenitor phenotype onto tumor-reactive T cells, and that *TCF7* expression is merely a readout for FOXO1 activity in mice and humans. Indeed, FOXO1_OE_ DEGs are enriched in a subset of *ex vivo* patient TILs that correlated with TIL therapy clinical responses, whereas TCF_OE_ DEGs are de-enriched. Surface markers and TFs that are often co-expressed in *TCF7*^*+*^ cells are FOXO1 target genes (e.g. *SELL*, *IL7R*, *KLF2*, and *MYB*^30^) and our empiric FOXO1 regulon significantly correlates with *TCF7* expression and clinical responses in patient CAR T samples, further supporting this notion. Conditional deletion of *Foxo1* in mature mouse T cells diminished the frequency of *Tcf7*-expressing T_PEX_^19^, raising the prospect that FOXO1 promotes cell states that are normally associated with high *Tcf7* expression. Since FOXO1 activity is regulated at the post-translational level rather than through changes in transcript^45,50^ and is therefore veiled in RNA-sequencing data, its role in cancer immunology and immunotherapy has likely been vastly underappreciated. Future mechanistic studies are warranted to determine the precise functional role of FOXO1 and TCF1 in human engineered and non-engineered T cells during cancer immunotherapy.

In summary, we demonstrate that FOXO1 is a master regulator of human T cell memory that can be leveraged to enhance the persistence and potency of CAR T cells. Our results suggest that FOXO1 represents a major therapeutic axis in T cell-based cancer immunotherapies and challenge the notion that TCF1 plays a critical role in CAR T cell responses. More broadly, our study provides evidence that pioneer transcription factors can enforce epigenetic and transcriptional programs that rewire T cell states and promote synthetic phenotypes, thereby paving the way for next-generation transcription factor engineering for cell therapies.

## METHODS

### Primary human T cells

For experiments completed at Stanford, anonymous healthy donor buffy coats were obtained from the Stanford University Blood Center (Stanford, CA) under a University Institutional Review Board-exempt protocol or obtained from Human Peripheral Blood Leukopak (Stemcell Technologies). CD3+ cells were isolated using the RosetteSep Human T Cell Enrichment Kit, Lymphoprep density gradient medium, and SepMate-50 tubes according to the manufacturer’s protocol (Stem Cell Technologies). For experiments completed at Children’s Hospital of Philadelphia, purified CD3+ healthy donor T cells were obtained from the University of Pennsylvania Human Immunology Core (Philadelphia, PA). All purified T cells were cryopreserved in CryoStor CS10 medium (Stem Cell Technologies).

### Cell lines

Cell lines were obtained from American Type Culture Collection (ATCC) and stably transduced to express markers as follows: 143B osteosarcoma cells express GFP and firefly luciferase, with or without CD19 (143B and 143B-19+, respectively). Nalm6 B-ALL cells express GFP and firefly luciferase, with or without GD2 (Nalm6 and Nalm6-GD2+, respectively). Single cell clones were chosen for high antigen expression. 143B and Nalm6 cells were cultured in DMEM and RPMI 1640, respectively, and both were supplemented with 10% FBS, 10mM HEPES, and 1x Penicillin-Streptomycin-Glutamate (Gibco).

### CAR and transcription factor construct design

CAR constructs used in this study include CD19.28ζ, CD19.BBζ, anti-GD2 HA.28ζ and Her2.BBζ. Codon-optimized TCF1, FOXO1, or FOXO1_3A_ sequences and a P2A ribosomal skip sequence were generated as Gene Blocks by IDT and constructed in an MSGV retroviral vectors. The tNGFR-only construct does not contain a P2A ribosomal skip sequence. The FOXO1_DBD_ construct was generated via 2-step mutagenic NEBuilder HiFi DNA Assembly (New England BioLabs). All plasmids were amplified by transformation into Stellar Competent *E. coli* (Takara Bio) and sequences were validated by sequencing (Elim Biopharmaceuticals).

### Retrovirus production

To generate retrovirus, 10 million 293GP cells were plated on a 15-cm BioCoat Poly-D-Lysine cell culture plate (Corning) and fed with 20mL of DMEM supplemented with 10% FBS, 10mM HEPES, and 1x Penicillin-Streptomycin-Glutamate (Gibco) 24 hours prior to transfection. Transfection was performed by mixing a room temperature solution of 3.4 mL Opti-MEM (Gibco) + 135uL Lipofectamine 2000 (Invitrogen) (solution 1) with a second solution of 3.4 mL Opti-MEM + 11 ug RD114 packaging plasmid DNA + 22ug MSGV retroviral plasmid of interest (solution 2) via slow dropwise addition of solution 2 to solution 1. The combined solution 1 and 2 mixture was incubated for 30 minutes at room temperature, after which medium was replaced on 293GP cells, and 6.5mL of the combined solution was added to the plates in a slow, drop-wise fashion. The next day, culture medium was replaced on 293GP cells. At 48 hours post-transfection, viral supernatant was harvested from the cells and culture medium was replaced; supernatant collection was repeated at 72 hours. At each harvesting step, supernatant was spun down to remove cells and debris, and frozen at −80°C for future use.

### T cell activation and culture

T cells were thawed in warm water after removal from liquid nitrogen and then washed with T cell medium (AIM-V [Gibco] supplemented with 5% fetal bovine serum (FBS), 10mM HEPES, 1x Penicillin-Streptomycin-Glutamate, and 100U/mL recombinant human IL-2 [Peprotech] or RPMI [Gibco] supplemented with 10% FBS, 10mM HEPES, 1x Penicillin-Streptomycin-Glutamate, and 100U/mL recombinant human IL-2). Human T-Expander αCD3/CD28 Dynabeads (Gibco) were washed and added to T cells at a volume of 30uL resuspended beads per million T cells. T cells and beads were then resuspended at a concentration of 500,000 T cells/mL in T cell medium (D0 for all assays). 48- and 72-hours post-activation, T cells were transduced (see “Retroviral Transduction,” below). 96 hours post-activation, beads were removed via magnetic separation using a DynaMag column (Invitrogen). T cells were fed with fresh T cell medium every 48–72 hours and maintained at a density of 0.5 × 10^6^ cells/mL post-feed. For FOXO1_i_ experiments, T cells were provided fresh complete T cell medium and vehicle control (DMSO) or AS1842856 (EMD Millipore) every 2–3 days from days 4 to 15 post-activation.

### Retroviral transduction

T cells were transduced with retrovirus on days 2 and 3 post-activation for all experiments. In brief, 12- or 24-well, non-tissue-culture-treated plates were coated with 1mL or 500uL, respectively, of 25ug/mL Retronectin (Takara) in PBS and placed at 4C overnight. The next day, plates were washed with PBS then blocked with 2% BSA + PBS for 10 minutes. Retroviral supernatants were added and plates were centrifuged at 32 °C for 2 hours at 2500 RCF. Viral supernatants were subsequently removed and T cells were added to each virus-coated well at a density of 1 × 10^6^ T cells/well for 12-well plates and 0.5 × 10^6^ T cells/well for 24-well plates.

### Cell selection

tNGFR isolations were performed using either Miltenyi MACS sorting or StemCell EasySep sorting unless otherwise stated. For Miltenyi MACS sorting, cells were resuspended in FACS buffer and stained with Biotin anti-human CD271 (tNGFR) antibody (BioLegend). Cells were washed with PBS, 0.5% BSA, and 2mM EDTA (MACS buffer), resuspended in MACS buffer and mixed with Streptavidin MicroBeads (Miltenyi), then washed again with MACS buffer and passed through an LS Column for positive selection inside a MACS separator (Miltenyi). For Stem Cell EasySep sorting, cells were isolated using the manufacturer’s protocol for the EasySep Human CD271 Positive Selection Kit II (StemCell) with an EasyEights EasySep Magnet (StemCell). After isolation, cells were immediately mixed with warm complete T cell media, counted, and resuspended at 500,000/mL.

For RNA-seq experiments on FOXO1_KO_ cells, CD62L_lo_CAR^+^ cells were isolated by negative selection by first staining cells with anti-CD62L-PE and then by following the EasySep PE Positive Selection Kit II protocol according to the manufacturer’s instructions (Stem Cell Technologies). For RNA and ATAC-seq experiments on tNGFR, TCF1_OE_ and FOXO1_OE_ cells, CD8^+^ tNGFR^+^ CAR T cells were isolated prior to sequencing using the EasySep Human CD8+ T Cell Isolation Kit (StemCell). For *in vivo* analysis of tumor-infiltrating CAR T cells, CD45^+^ T cells were isolated from tumors using the EasySep Release Human CD45 Positive Selection Kit (Stem Cell Technologies) according to the manufacturer’s instructions.

### CRISPR/Cas9 gene editing

To interrogate the role of endogenous FOXO1 on CAR T cell function, CRISPR-Cas9 was used to delete a sequence directly upstream of the *FOXO1* DNA binding domain. On day 4 post-activation, retrovirally-transduced CAR T cells were removed from activation beads by magnetic separation. 20 μL reactions were prepared by resuspending 1 million CAR T cells in P3 buffer immediately prior to electroporation with the P3 Primary Cell 4D-Nucleofector Kit (Lonza). Ribonucleoproteins were prepared by complexing 0.15ng of sgRNA targeting *FOXO1* or *AAVS1* (Synthego) with 5 μg Alt-R S.p. Cas9 Nuclease (IDT cat# 1081058) prior to adding the cell suspension to each reaction. For *AAVS1* edits, a previously validated sgRNA sequence (5’ GGGGCCACUAGGGACAGGAU 3’) was used. For *FOXO1*, two sgRNAs were used in tandem at equal concentrations (5’ UUGCGCGGCUGCCCCGCGAG 3’ and 5’ GAGCUUGCUGGAGGAGAGCG3’). The reaction was pulsed with the EH115 program on a Lonza 4D Nucleofector. Cells were recovered immediately in 260 μl of warm complete AIM-V media supplemented with 500 U/mL IL-2 in round bottom 96 well plates, and expanded into 1 mL fresh medium after 24 hours. Cells were maintained at densities of 0.5 to 2 million cells per mL in well plates until day 14–16 for functional and phenotypic characterization. On days 14–16, knockout efficiency was determined by intracellular transcription factor staining (Cell Signaling cat# 58223) followed by flow cytometry.

### Flow cytometry

CAR T cells were washed twice in FACS buffer (PBS + 2% FBS) and stained with fluorophore-conjugated surface antibodies for 30 minutes on ice. Cells were washed twice with FACS buffer prior to analysis. Intracellular stains were performed with the same initial surface stain, after which cells were fixed, permeabilized, and stained using the FoxP3 Transcription Factor Staining Buffer Set according to the manufacturer’s protocol (eBioscience). 1A7 anti-14G2a idiotype antibody used to detect the HA CAR was obtained from the NCI and conjugated using the Dylight 650 antibody labeling kit (Thermo Fisher). The anti-FMC63 idiotype antibody was manufactured by GenScript and fluorescently conjugated using Dylight 650 antibody labeling kit. Cells analyzed with either a BD Fortessa running FACS Diva Software, or a Cytek Aurora using SpectroFlo Software. Downstream analyses were performed using FlowJo v. 10.8.1 Software. All reagents are listed in Table 2.

### Cytokine secretion assays

5 × 10^4^ CAR T cells were co-cultured with 5 × 10^4^ tumor cells in 200 uL of complete T cell medium (AIM-V or RPMI) without IL-2 in a 96-well plate, all in triplicate. 24 hours after coculture, culture supernatants were collected, diluted 20 to 100-fold and analyzed for IL-2 and IFN*γ* using ELISA MAX kits (BioLegend) and Nunc Maxisorp 96-well ELISA plates (Thermo Scientific). Absorbance readings were collected on a Tecan Spark plate reader. For FOXO1_i_ assays, co-culture medium included concentrations of AS1842856 that were used during T cell expansion.

### Incucyte killing assay

5 × 10^4^ GFP+ tumor cells and T cells corresponding to a 1:1, 1:2, 1:4, 1:8, and/or 1:16 effector:target ratios were co-cultured in 300 uL of T cell medium without IL-2 in 96-well flat-bottom plates. Plates were imaged at 10X zoom with 4–9 images per well every 2–4 hours for 96 hours using the IncuCyte ZOOM Live-Cell analysis system (Essen BioScience/Sartorius). Total integrated GFP intensity per well or total GFP area (um^2^/well) were used to analyze expansion or contraction of Nalm6 or 143B cells, respectively. All GFP intensity/area values were normalized to the first imaging time point (*t* = 0). For FOXO1_i_ assays, co-culture medium included concentrations of AS1842856 that were used during T cell expansion.

### Repeat stimulation assay

CAR T cells were activated, transduced, and tNGFR+ cells were isolated as described above. Cells were cultured in AIM-V with IL-2 until day 14 “pre-stim” assays, including flow cytometry, cytokine secretion and Incucyte as described above. On day 14, co-cultures were set up comprising of 5 × 10^5^ T cells and 2 × 10^6^ Nalm6 tumor cells suspended in AIM-V without IL-2 at a final concentration of 5 × 10^5^ total cells per mL. Cocultures were fed with 5mL of AIM-V without IL-2 on day 3 of culture. On day 3 of the repeat stim co-culture, CAR T cells were again assayed via cytokine secretion, Incucyte killing assay, flow cytometry as described above. This process was repeated for 4 total co-cultures such that cytokine and Incucyte assays were set up for four serial stimulations on days 14, 17, 20, and 23 on cells that had been stimulated with Nalm6 tumor zero, one, two, and three previous times, respectively for a total of four serial stimulations by the end of the experiment. Cells were analyzed via flow cytometry on day 7 of co-culture, such that T cells were co-cultured with tumor on days 14, 17, 20, and 23 and analyzed on days 21, 24, 27, and 30, respectively.

### Seahorse Assay

Metabolic analyses were carried out using Seahorse Bioscience Analyzer XFe96. In brief, 0.2 × 10^6^ cells were resuspended in extracellular flux (XF) assay media supplemented with 11 mM glucose, 2 mM glutamine, and 1 mM sodium pyruvate and plated on a Cell-Tak (Corning)–coated microplate allowing the adhesion of CAR T cells. Mitochondrial activity and glycolytic parameters were measured by the oxygen consumption rate (OCR) (pmol/min) and extracellular acidification rate (ECAR) (mpH/min), respectively, with use of real-time injections of oligomycin (1.5 M), carbonyl cyanide ptrifluoromethoxyphenylhydrazone (FCCP; 0.5 M), and rotenone and antimycin (both at 0.5M). Respiratory parameters were calculated according to manufacturer’s instructions (Seahorse Bioscience). Reagent sources are listed in Table 2.

### Immunoblotting

Chromatin-bound and soluble proteins were separated as previously described^24^. Briefly, cytoskeletal (CSK) buffer was prepared using 100mM NaCl, 300mM sucrose, 3mM MgCl2, 10mM PIPES (pH 6.8), 0.1% IGEPAL CA-630, 4μg/mL aprotinin, 10μg/mL leupeptin, 4μg/mL pepstatin, and 2mM PMSF. After washing with ice-cold PBS, cell pellets were lysed with CSK buffer for 20 minutes on ice. Samples were centrifuged at 1500 RCF for 5 min and the soluble fraction was separated and cleared by centrifugation at 15870 RCF for 10 min. The protein concentration of the soluble fraction was determined by DC protein assay (Bio-Rad, Cat#5000116). The remaining pellet containing the chromatin-bound fraction was washed twice with CSK buffer, centrifuging at 1500 RCF for 5 min. Chromatin-bound proteins were resuspended in CSK buffer and 1X Pierce Reducing Sample Buffer (Thermo Scientific, Cat#39000) and boiled for 5 min for solubilization. The soluble fraction was supplemented with Pierce Reducing Sample Buffer to achieve 1X and boiled for 5 min. For immunoblotting, equal amounts of soluble and chromatin-bound fraction for each sample were analyzed by SDS-polyacrylamide gel electrophoresis and transferred to nitrocellulose membranes (Bio-Rad, Cat#1704158). Membranes were blocked for 30 min in 5% milk in TBST (1X Tris-buffered saline containing 0.1% Tween 20). After washing with TBST, membranes were incubated with anti-FOXO1 antibody (1:1000; Cell Signaling, #2880, clone C29H4) overnight at 4°C. Next, membranes were washed with TBST and incubated with anti-mouse (1:10,000, Cell Signaling, #7074) or anti-rabbit (1:10,000, Cell Signaling, #7076) IgG conjugated to horseradish peroxidase for 1 hr at RT. Membranes were visualized using Clarity Western ECL Substrate (Bio-Rad, Cat#1705060) and the ChemiDoc Imaging System (Bio-Rad). After visualization, membranes were stripped using a mild stripping buffer (1.5% glycine, 0.1% SDS, 1% Tween 20, pH 2.2). The previous steps were repeated for detection of soluble (1:5000 GAPDH; Cell Signaling, #97166, clone D4C6R) and chromatin-bound (1:1000 Lamin A; Cell Signaling, #86846, clone 133A2) fraction loading controls.

### Murine xenograft models

NOD/SCID/IL2Rγ^−/−^ (NSG) mice were bred, housed, and treated under Stanford University APLAC- or Children’s Hospital of Philadelphia (CHOP) ACUP-approved protocols. 6–8 week-old mice were healthy, immunocompromised, drug- and test-naïve, and unused in other procedures. Mice were housed at the Stanford Veterinary Service Center (VSC) or CHOP Department of Veterinary Services (DVR) in a barrier facility with a 12-hour light/dark cycle. Mice were monitored daily by VSC or DVR staff and euthanized if endpoint criteria were met, including hind limb paralysis, rough coat, impaired mobility, hunched posture, excessive cachexia, and tumor sizes exceeding animal protocol limits. In Nalm6-bearing mice, 2 × 10^5^ to 1 × 10^7^ cells in 100–200 uL of sterile PBS were engrafted via tail vein injection (TVI). In 143B osteosarcoma models, 1× 10^6^ to 3 × 10^6^ cells in 100uL sterile PBS were engrafted via intramuscular injection into the flank. CAR T cells were engrafted via TVI at doses and schedules noted in the main text. Nalm6 engraftment, expansion, and clearance were measured by intraperitoneal injection of luciferin and subsequent imaging via a Spectrum IVIS bioluminescence imager and quantified using Living Image software (Perkin Elmer) or via a Lago X imager and quantified using Aura software (Spectral Instruments Imaging), all under isoflurane anesthesia. 143B tumor size was monitored via caliper measurements.

### Murine tissue analyses

Peripheral blood was sampled from live, isoflurane-anesthetized mice via retro-orbital blood collection. 50uL of blood was labeled with surface antibodies, lysed using FACS Lysing Solution (BD), and quantified using CountBright Absolute Counting Beads (Thermo Fisher) then analyzed on a BD Fortessa cytometer. For phenotypic analysis of spleen and tumors, mice were euthanized and tissues were mechanically dissociated and washed twice in PBS. Spleens were placed in a 6 cm petri dish and filtered through a sterile 70 μm cell strainer. Tumors were mechanically and chemically dissociated with Collagenase IV and DNAse in HBSS and incubated at 37°C with shaking for 30 min. Cells were mashed through a sterile 70 μm cell strainer before washing with PBS. Cells from both spleens and tumors were spun down at 450 RCF for 5 min at 4°C, then treated with ACK lysis buffer for 3 min on ice. Cell suspensions were washed twice with PBS and CAR T cells were isolated by positive selection using the EasySep Release Human CD45 Positive Selection Kit. Cells were stained for markers of interest and analyzed on a Cytek Aurora using SpectroFlo Software.

### RNA-seq

0.5–1 × 10^6^ T cells were pelleted by centrifugation and flash frozen. Pellets were thawed on ice and processed using either a RNEasy Plus Mini Kit or an AllPrep DNA/RNA Micro Kit (for simultaneous DNA and RNA isolation) (Qiagen) according to the manufacturer’s instructions. Total RNA was quantified using either a Qubit Fluorometer or a DeNovix DS-11 FX Spectrophotometer/Fluorometer and sequenced using 150bp paired end read length and ~50 million read pairs per sample (Novogene).

### RNA-seq processing and analysis

We processed the sequencing data using the nf-core RNA-seq pipeline (https://nf-co.re/rnaseq). In brief, we performed quality control of the fastq files using FastQC and trimmed the filtered reads with Trim Galore software. The trimmed fastq files resulting from the experiment were aligned to the hg38 human genome using STAR. Salmon was then used to generate a gene-by-sample count matrix for downstream analysis. PCA was performed on read counts that were processed using the variance-stabilizing transformation. To correct for batch effects by donor, the removeBatchEffect function in the limma package was utilized. Differential analysis of gene expression was conducted using the DESeq2 package, with an absolute log2 fold change of >= 0.5 and FDR < 0.05. To create a heatmap, differential genes were aggregated, and expressions were standardized with z-scores across samples. The k-means clustering algorithm with Pearson correlation as the distance metric was used to cluster the genes. Pathway analysis of the differential genes and grouped genes in the heatmap was performed using QIAGEN Ingenuity Pathway Analysis and clusterProfiler. Cell-type enrichment was performed through the single-sample extension of Gene Set Enrichment Analysis (ssGSEA) in the GSVA R package^65^ using signature genes from Andreatta et al.^66^ and Krishna et al.^8^

### Bulk ATAC-seq processing

CD8^+^ tNGFR^+^ CAR T cells were isolated using the EasySep Human CD8+ T Cell Isolation Kit. 150,000 CD8+ T cells were slow-frozen in BamBanker (Bulldog Bio) cell preservation media. Approximately 100k CAR-T cells were washed in ice-cold PBS and subjected for nuclei isolation using the following lysis buffer: 10mM Tris-HCl pH 7.5, 10mM NaCl, 3mM MgCl2, 0.1% Tween-20, 0.1% NP40, 0.01% Digitonin and 1% BSA. After washing the cells, 50 ul lysis buffer was added to each sample and cells were resuspended by pipetting. Nuclear pellets were centrifuged and resuspended in the transposase reaction containing 10.5ul H_2_O, 12.5ul 2xTD buffer and 2ul Tn5 transposase in total of 25ul. The reaction was incubated for 30 minutes at 37°C. The reaction was stopped by the addition of 75ul TE buffer and 500ul PB buffer (Qiagen), followed by column purification per manufacturer’s recommendation (Qiagen, Minelute Kit). DNA was eluted from the columns in 22ul H_2_O. PCR reactions were set up as follows: 21 ul DNA, 25 ul Phusion master mix (NEB) and 2 ul of each barcoded PCR primer (ApexBio, K1058). 15 PCR cycles were run for each sample. Reactions were cleaned up with AMPure XP beads according to the recommendations of the manufacturer. Libraries were quantified with Qubit fluorometer and fragment analysis was performed with Bioanalyzer. Libraries were sequenced on a NovaSeq 6000 sequencer.

### Bulk ATAC-seq analysis

ATAC-seq libraries were processed using the pepatac pipeline (http://pepatac.databio.org/) with default options. In brief, fastq files were trimmed to remove adapter sequences, and then pre-aligned to the mitochondrial genome to exclude mitochondrial reads. To ensure the accuracy of downstream analysis, multimapping reads aligning to repetitive regions of the genome were filtered from the dataset. Bowtie2 was then used to align the reads to the hg38 genome. Samtools was employed to identify uniquely aligned reads, and Picard was used to remove duplicate reads. The resulting deduplicated and aligned BAM file was used for downstream analysis. Peaks in individual samples were identified using MACS2 and compiled into a non-overlapping 500 bp consensus peak set. Briefly, the peaks were resized to 500bp-width and ranked by significance. The peaks that overlapped with the same region were selected by ranks and the most significant peak was retained. The peak-sample count matrix was generated using ChrAccR with the default parameters of the run_atac function. Signal tracks for individual samples were generated within the pepatac pipeline. These tracks were then merged by group using WiggleTools to produce a comprehensive view of the data across all samples.

Based on our analysis of the peak-sample count matrix, the DESeq2 package was used to identify differential peaks across different conditions, with a threshold of an absolute log2 fold change greater than 0.5 and an FDR less than 0.05. To generate PCA plots, we first extracted a variance-stabilized count matrix using the vst function in DESeq2. Next, we corrected for batch effects by donor using the removeBatchEffect function in the limma library. Finally, we generated PCA plots using the corrected matrix with the plotPCA function. We aggregated differential peaks across conditions, standardized the peak signals using z-scores across samples, and performed k-means clustering to generate a chromatin accessibility heatmap. Motif enrichments of differential peaks and grouped peaks were searched with HOMER and findMotifsGenome.pl with default parameters. The enrichment of cell-type specific regulatory elements are performed with the gchromVAR package^67^. Briefly, this method weights chromatin features by log2 fold changes of cell-type specific regulatory elements from Satpathy et al.^9^ and computes the enrichment for each cell type versus an empirical background matched for GC content and feature intensity.

### FOXO1 Regulon identification and analysis

The FOXO1 regulon gene set was generated by intersecting down-regulated differential genes (log2 fold change < −0.25, FDR < 0.05) in FOXO1_KO_ cells and up-regulated differential genes (log2 fold change > 0.5, FDR < 0.05) in FOXO1_OE_ cells (Table 1). Regulon enrichment scores were calculated using the single-sample extension of Gene Set Enrichment Analysis (ssGSEA) in the GSVA R package on the Fraietta et al. RNA expression dataset^3^.

For regulon analyses of scATAC-seq data, the processed Signac data object of CAR T products profiled via scATAC-seq were obtained from Chen et al.^6^. To account for sample-to-sample variability, the mean fragments in peaks per cell were downsampled for consistency between donors. Further, donors PT48 and PT51 were excluded based on low data quality after examination of quality control statistics, including per-library transcription start site enrichment. Using the epigenetic signature for FOXO1 and TCF1 over expression (Figure 4), we computed the per-cell epigenetic signature per factor using the chromVAR workflow as previously described^68^ for related T cell signatures derived from bulk experiments. To test for differences in responder / non-responder associations with this signature, we performed an ordinary least squares regression with the per-cell z-score against the donor’s B cell aplasia status at 6 months, adjusting for individual patient ID. Statistical significance was based on the Wald Test statistic of the coefficient for the responder term in the two regressions for each factor.

For regulon analyses of the CLL CD19 CAR T cell clinical dataset, the gene expression data table for CLL patient activated CD19 CAR T-cell products was obtained from Joseph A Fraietta et al. The enrichment of FOXO1 signature was analyzed using the single-sample extension of Gene Set Enrichment Analysis (ssGSEA) as previously described^69,70^, and carried out using the R package GSVA v1.46.0. To compare the ssGSEA enrichment scores between responders and nonresponders, a Mann-Whiteney test was conducted. To statistically determine optimal stratification points for survival analysis, we compared candidate stratification points based on hazard ratio and P value as previously described. The survival analysis was conducted with a Log-rank (Mantel-Cox) test using Prism v.9.5.0.

### Statistical Analyses

Unless otherwise stated, statistics analyses for significant differences between groups were conducted using one- or two-way analysis of variance (ANOVA) with Bonferroni or Dunnett multiple comparisons test, or with a Student’s or Welch’s t test using GraphPad Prism v. 9.4.1. In experiments where same-donor samples were compared across two conditions, we performed a paired Student’s t test. Experiments where data were measured at zero or below the limit of detection were excluded unless otherwise stated. Survival curves were compared using the log-rank Mantel-Cox test.

## Extended Data

## Data Availability

All data associated with this paper are included in the manuscript and the supplementary materials. RNA- and ATAC-seq data will be deposited to the Gene Expression Omnibus upon publication of the manuscript. All code associated with this paper will be deposited to the Weber Lab GitHub page upon publication.
